# IRF5 Mediates Artery Inflammation in Salt-Sensitive Hypertension by Regulating STAT1 and STAT2 Phosphorylation to Increase ESM1 Transcription: Insights from Bioinformatics and Mechanistic Analysis

**DOI:** 10.3390/ijms26083722

**Published:** 2025-04-15

**Authors:** Qiaoyu Shao, Hao Wang, Shicheng Li, Mengying Zeng, Shuyang Zhang, Xiaowei Yan

**Affiliations:** 1 Department of Cardiology, Peking Union Medical College Hospital, Chinese Academy of Medical Sciences & Peking Union Medical College, Beijing 100730, China; shaoqy@163.com (Q.S.); tabblk@163.com (H.W.); leesc_gx@foxmail.com (S.L.); kjyzmy1924@163.com (M.Z.); 2 Department of Medicine, Brigham and Women’s Hospital, Harvard Medical School, Boston, MA 02115, USA; 3 Medical Research Center, State Key Laboratory of Complex Severe and Rare Diseases, Chinese Academy of Medical Sciences & Peking Union Medical College Hospital, Beijing 100730, China

**Keywords:** salt-sensitive hypertension, artery inflammation, IRF5, STAT1, STAT2, ESM1

## Abstract

Salt-sensitive hypertension (SSH) is closely associated with arterial inflammation, yet its molecular mechanisms remain unclear. In this study, we utilized deoxycorticosterone acetate (DOCA)-salt-induced hypertensive mice, which exhibited elevated blood pressure and significant arterial inflammation. Single-cell RNA sequencing (scRNA-seq) identified interferon regulatory factor 5 (IRF5) and its downstream targets, signal transducer and activator of transcription (STAT), as key regulators of these inflammatory changes. In vivo, IRF5 levels were significantly elevated in the DOCA group, while STAT1 and STAT2 protein levels were comparable to those in the normal salt group. However, nuclear levels of phosphorylated STAT1 (pSTAT1) and phosphorylated STAT2 (pSTAT2) were markedly higher in the DOCA group. Furthermore, scRNA-seq analysis showed increased IRF5 expression in endothelial cells (ECs) in both human and mouse aorta samples. In vitro, IRF5 knockdown in artery ECs led to a reduction in nuclear pSTAT1 and pSTAT2 expression. These results suggest that IRF5 promotes STAT1 and STAT2 phosphorylation, enabling their nuclear translocation. Additionally, RNA sequencing indicated a positive correlation between endothelial cell-specific molecule 1 (ESM1) and STAT1/STAT2. Using the UCSC and JASPAR databases, we identified multiple binding sites for the STAT1::STAT2 dimer on the ESM1 promoter. Luciferase reporter assays revealed enhanced ESM1 transcription following pSTAT1::pSTAT2 binding, and pinpoint potential binding sites. Chromatin Immunoprecipitation Quantitative PCR (ChIP-qPCR) further confirmed the specific binding sites between the pSTAT1::pSTAT2 dimer and the ESM1 promoter. These findings highlight the critical role of the IRF5-pSTAT1::pSTAT2-ESM1 pathway in the pathogenesis of SSH and suggest potential therapeutic targets.

## 1. Introduction

Hypertension is a widespread cardiovascular disorder and a major global health issue, as high blood pressure (BP) is a key risk factor for cardiovascular events like stroke and myocardial infarction, as well as renal complications [1,2,3]. Among the various types of hypertension, salt-sensitive hypertension (SSH) is characterized by changes in BP in response to salt intake, defined as an increase in BP of 5% to 10% or at least 5 mmHg following elevated sodium intake [4,5,6]. Salt sensitivity is present in both normotensive and hypertensive people, affecting about 25% of the general population and over 50% of those with hypertension, who have enhanced susceptibility to SSH [7,8,9,10]. Salt sensitivity is influenced by physiological, environmental, genetic, and demographic factors [7,11], with post-menopausal women, African Americans, and the elderly being particularly susceptible [12,13]. Despite extensive research, the biological mechanisms underlying salt sensitivity and SSH remain poorly understood.

Evidence increasingly points to chronic, low-grade inflammation as a critical factor in the development and progression of hypertension, especially in those sensitive to salt [14,15,16,17]. High sodium acts as a potent trigger for inflammatory activation, leading to vascular dysfunction and hypertension in experimental animals [18,19], while immune suppression has been shown to attenuate hypertension in these models [20,21,22,23]. Clinical studies further support this link, showing that inflammation markers are associated with elevated BP [24,25], while anti-inflammatory treatments have demonstrated the potential to lower BP [26]. Salt sensitivity-induced vascular dysfunction and inflammation affect not only small resistance arteries, which manifest as microvascular endothelial inflammation, anatomic remodeling, and functional abnormalities [27], but they also impact the function and structure of large elastic arteries [28]. These findings suggest that salt-induced vascular changes extend throughout the arterial system. Therefore, addressing arterial inflammation and remodeling is essential for understanding the pathogenesis of SSH and developing targeted strategies to manage its cardiovascular complications.

Several previous studies have demonstrated that interferon regulatory factor (IRF)-5 is involved in numerous diseases, including atherosclerosis, systemic lupus erythematosus, rheumatoid arthritis, inflammatory bowel diseases, and periodontitis [29,30,31,32,33]. Among the ten transcription factors in the IRF family, IRF5 may play the most important role in inflammation-driven diseases. Indeed, IRF5 regulates type I interferons (Interferon-α/β) and initiates the expression of several pro-inflammatory cytokines, such as TNF-α, IL-12, and IL-6 [34,35,36]. IRF5 also acts as a molecular switch in macrophage-mediated inflammatory pathways [36] and plays a critical role in endothelial inflammation. Its expression is rapidly induced by ischemia–reperfusion injury, promoting leukocyte adhesion to ECs via the Janus kinase 2 (JAK2)-signal transducer and activator of transcription 3 (STAT3) pathway by upregulating vascular cell adhesion molecule-1 (VCAM-1) expression [37]. Interestingly, IRF4, another member of the IRF family, plays a crucial role in the pathogenesis of hypertension-induced renal inflammation and fibrosis, as demonstrated by reduced renal dysfunction and fibrotic response in IRF4 knockout mice subjected to DOCA treatment [38]. However, little is known regarding the physiological function of IRF5 in endothelial dysfunction in salt-sensitive hypertension.

The signal transducer and activator of transcription (STAT) family serves as a central mediator of inflammatory cytokine signal transduction and plays a crucial role in regulating immune cell differentiation and function [39]. In a resting state, STAT proteins exist in an inactive form in the cytoplasm. Upon extracellular stimulation, such as cytokines or growth factors activating their respective receptors, the tyrosine residues of STAT proteins undergo phosphorylation, leading to the formation of homo- or heterodimers. These dimers then translocate into the nucleus, where they directly regulate the transcription of target genes [40,41]. Notably, existing studies suggest that the functional activation of STAT proteins depends on the simultaneous processes of phosphorylation and dimerization. To date, there is no evidence indicating that monomeric tyrosine-phosphorylated STAT proteins possess biological activity [42]. Among the molecular subtypes, STAT1, STAT3, STAT4, STAT5A, and STAT5B can form homodimers, while STAT1/STAT2 and STAT1/STAT3 can form heterodimers [39]. Members of the STAT family participate in immune-inflammatory regulation by specifically mediating distinct cytokine signals: for example, STAT1/STAT2 primarily transmit IFN signals, STAT3 is responsible for transducing signals from IL-6 and other gp130 receptor ligands, while STAT4 and STAT6 are involved in IL-12 and IL-4 signaling, respectively. Basic research has confirmed the involvement of STAT1 and STAT3 in the pathogenesis of pulmonary arterial hypertension, with STAT1 activation being closely associated with a pro-inflammatory phenotype, whereas STAT3 promotes cell survival and inhibits apoptosis [43,44,45]. However, significant gaps remain in the understanding of the role of the STAT family in the pathophysiology of hypertension. The limited in vitro evidence available suggests that the JAK2/STAT3 pathway may be involved in the inflammatory response in SSH [46].

Endothelial cell-specific molecule 1 (ESM1), an EC-associated proteoglycan, is upregulated by proangiogenic and pro-inflammatory cytokines and plays an essential role in processes such as cell adhesion, migration, and proliferation. By inhibiting leukocyte adhesion to intercellular adhesion molecule-1 (ICAM1), ESM1 appears to mitigate leukocyte extravasation during inflammation [47]. ESM1 is also implicated in endothelial-dependent pathological conditions and is often elevated in conditions linked to endothelial impairment, serving as both a marker and mediator of endothelial dysfunction [48,49,50,51].

In this study, we identified aortic inflammation, including remodeling, fibrosis, and apoptosis, in a DOCA-induced SSH mouse model and investigated the role of IRF5 in regulating vascular inflammation, which points to a new direction for future immunotherapy in hypertension.

## 2. Results

### 2.1. Establishment of the SSH Mouse Model and Ultrasound Imaging

**Figure 1:**
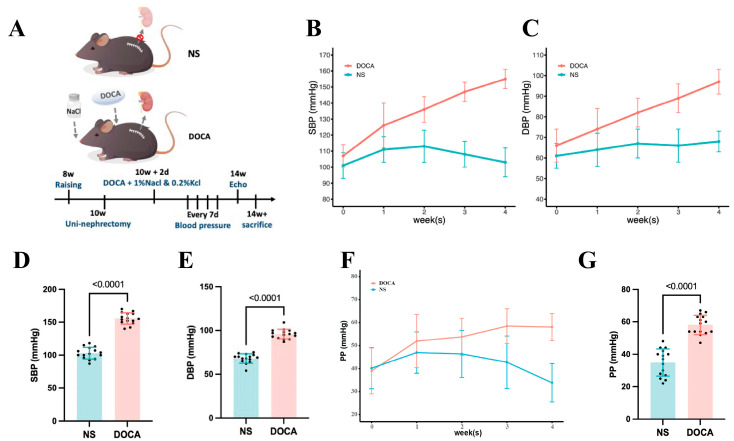
**Establishment and blood pressure assessment of the DOCA salt-sensitive hypertensive mouse model.** (**A**) The NS group (sham-operated) received tap water, while the DOCA group underwent unilateral nephrectomy followed by administration of deoxycorticosterone acetate (DOCA) along with water supplemented with 1% NaCl and 0.2% KCl. (**B**) Systolic blood pressure (SBP) and (**C**) diastolic blood pressure (DBP) in NS and DOCA groups measured from baseline to the fourth week. (**D**) Both SBP and (**E**) DBP showed a significant increase in the DOCA group by the fourth week, indicating successful establishment of the DOCA mouse model. (**F**) Pulse pressure (PP) in NS and DOCA groups measured from baseline to the fourth week. (**G**) PP showed a significant increase in the DOCA group by the fourth week, indicating arterial stiffness.

We established the SSH mouse model by administering DOCA and salt for 4 weeks (Figure 1A). SBP and DBP in the DOCA group continuously increased throughout the modeling period (Figure 1B,C). By week 4, SBP reached 155.0 ± 6.0 mmHg, significantly higher than in the NS group, with a corresponding increase in DBP (Figure 1D,E). Additionally, pulse pressure (PP) was significantly elevated in the DOCA group by the 4th week, indicating increased arterial stiffness (Figure 1F,G).

**Figure 2:**
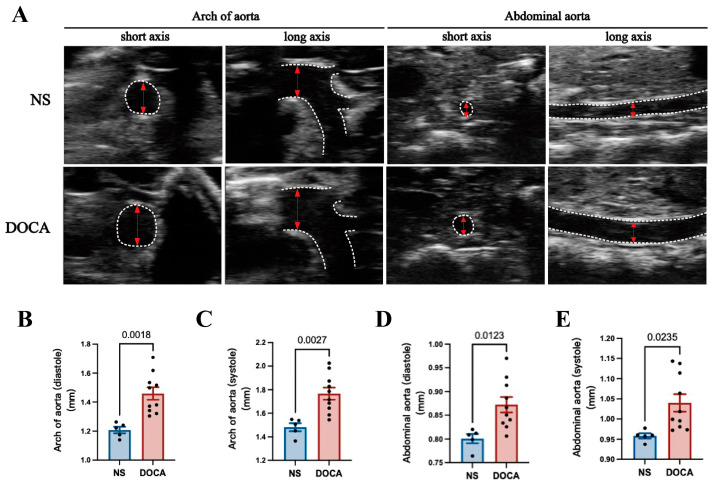
**Ultrasound imaging and quantification of aortic remodeling in DOCA salt-sensitive hypertension.** (**A**) Representative ultrasound images of the aortic arch and abdominal aorta during systole in both short and long axis views. (**B**,**C**) Comparison of the inner diameter (red arrows) of the aortic arch between the NS and DOCA groups during both (**B**) diastole and (**C**) systole. (**D**,**E**) Comparison of the inner diameter of the abdominal aorta between the NS and DOCA groups during both (**D**) diastole and (**E**) systole.

Ultrasound imaging revealed a significant increase in the aortic arch diameter in the DOCA group during both diastole and systole (Figure 2A–C). Similarly, the abdominal aorta showed notable enlargement in the DOCA group compared to the NS group (Figure 2A,D,E).

### 2.2. SSH Induces Artery Remodeling, Fibrosis, and Apoptosis

**Figure 3:**
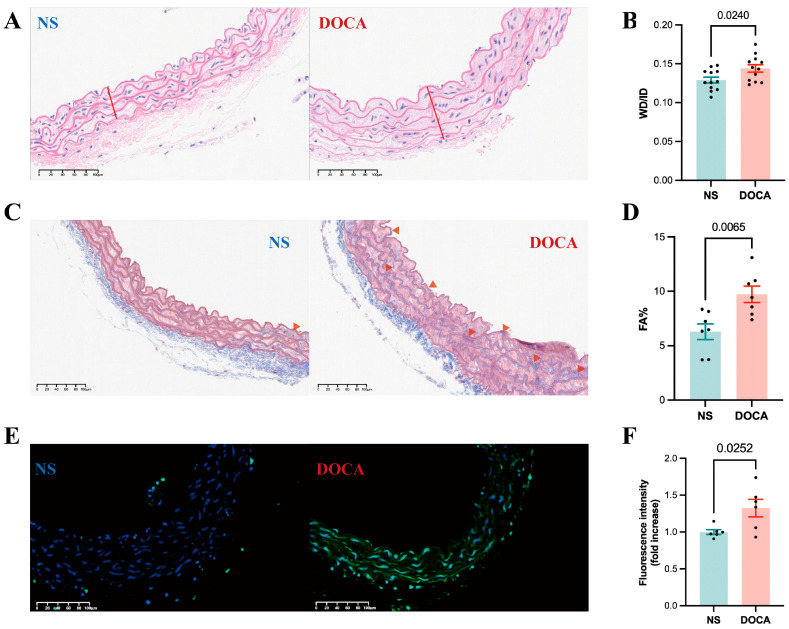
**Effects of DOCA on thoracic aortic vascular remodeling in DOCA salt-sensitive hypertension mice.** (**A**) Representative micrographs of mouse descending thoracic aorta stained by HE staining. (**B**) Wall thickness (red line) to inner diameter ratio from HE staining. (**C**) Representative micrographs of mouse descending thoracic aorta stained by Masson staining (red arrow). (**D**) Fibrotic area percentage from Masson staining. (**E**) Representative micrographs of mouse descending thoracic aorta stained by TUNEL staining. (**F**) Fluorescence intensity from TUNEL staining.

HE staining of descending thoracic aortic sections from the DOCA group revealed thickened vascular walls, irregular ECs arrangements, increased nuclear density, and disordered myofibril structure, all indicative of arterial remodeling (Figure 3A). Vascular remodeling parameters, such as the wall thickness to inner diameter ratio (WT/ID), were significantly higher in the DOCA group (Figure 3B), suggesting hypertrophic remodeling of the arteries. Masson staining further confirmed substantial collagen fiber deposition in the descending thoracic aorta of the DOCA group (Figure 3C), evidenced by a marked increase in the collagen volume fraction (Figure 3D). Additionally, the TUNEL assay revealed significantly higher fluorescence intensity in the descending thoracic aorta of the DOCA group compared to the NS group, indicating increased apoptosis in SSH (Figure 3E,F).

### 2.3. RNA Sequencing (RNA-Seq) Reveals Differential Gene Expression and Upregulation of Inflammatory Pathways

**Figure 4:**
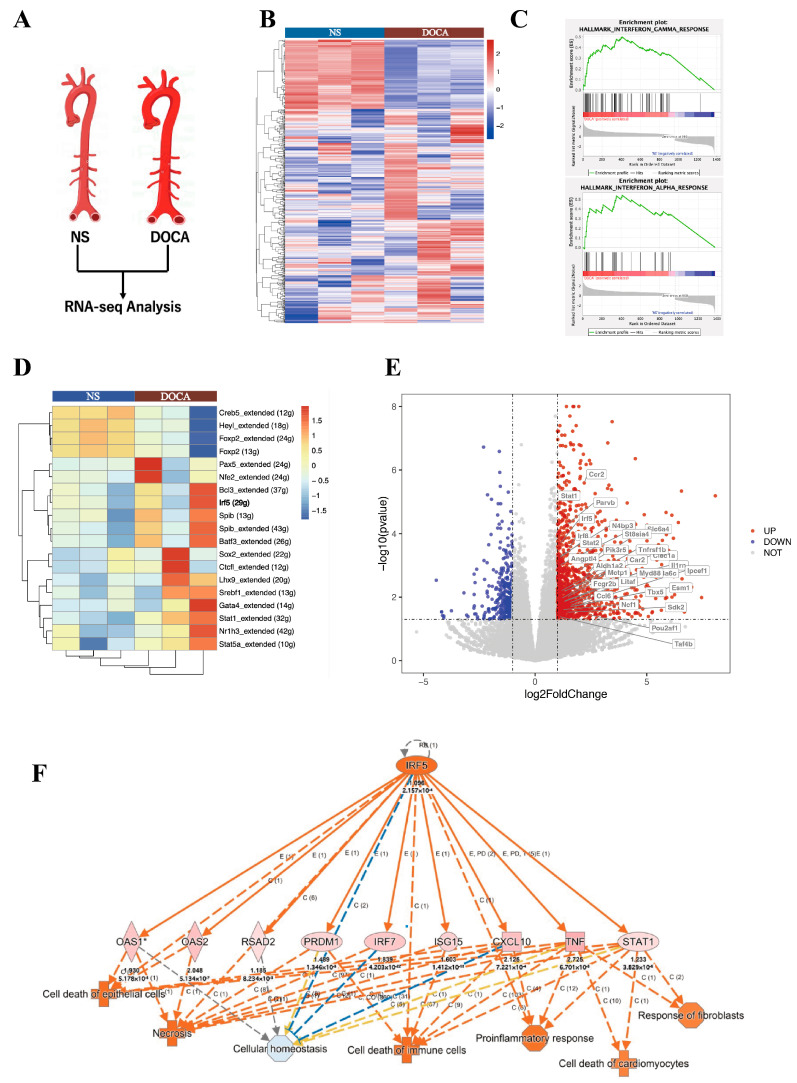
**RNA-seq and single-cell seq analysis of salt-sensitive hypertension.** (**A**) RNA-seq analysis comparing the NS group and the DOCA group. (**B**) Heat map of differentially expressed genes (DEGs). (**C**) Gene Set Enrichment Analysis (GSEA) shows DEGs enriched in the interferon-γ and interferon-α response pathways. (**D**) Single-cell regulatory network inference and clustering (SCENIC) analysis of DEGs identifies IRF5. (**E**) Volcano plot of all DEGs with IRF5 targets upregulated in the DOCA group. (**F**) Ingenuity Pathway Analysis (IPA) of IRF5 and its downstream targets. High salt induces IRF5 activation, leading to cell death and fibrosis through its downstream targets.

We applied RNA-Seq to examine transcriptional changes between the DOCA and NS groups in whole aorta tissues (Figure 4A). A total of 1434 differentially expressed genes (DEGs) were identified, with 1081 genes upregulated and 353 downregulated. Principal component analysis demonstrated a clear distinction between the DOCA and NS groups (Figure S1A). A heatmap visualizing all DEGs between the NS group and DOCA group (Figure 4B). To further investigate the biological functions of these DEGs, we conducted pathway enrichment analysis and Single-cell Regulatory Network Inference and Clustering (SCENIC) analysis. Gene Ontology (GO) pathway analysis further highlighted significant enrichment in immune and inflammatory response pathways (Sup Figure 1B). Gene Set Enrichment Analysis (GSEA) revealed strong enrichment of the interferon (IFN)-γ and IFN-α response pathways in the DOCA group. The IFN-γ pathway was associated with genes such as IRF5, STAT1, STAT2, and KLRK1 (Figure 4C and Figure S2A), while the IFN-α response pathway was enriched for genes such as STAT2, IFI44, and TAP1 (Figure 4C, Figure S2B). SCENIC analysis based on all DEGs identified upstream transcription factors with significantly higher regulatory activity in the DOCA group compared to the NS group (Figure 4D). Among these, IRF5 showed markedly elevated activity in the DOCA group, suggesting its pivotal role in upstream regulation or target gene activation under high-salt DOCA-induced conditions. STAT1 also displayed increased activity, albeit to a lesser extent, potentially reflecting a downstream effect.

A volcano plot of bulk RNA-seq highlighted the upregulation of IRF5, STAT1, and STAT2 in the DOCA group (Figure 4E). We further examined the linear relationship between IRF5 and STAT1 RNA levels in both NS and DOCA groups, revealing a strong positive correlation (Figure S2C). Given that STAT2 typically forms heterodimers with STAT1 to exert its functional activity and is enriched in the IFN-γ and IFN-α response pathways, we also analyzed its correlation with IRF5. Similarly, a strong positive correlation was observed between IRF5 and STAT2 transcript levels (Figure S2D). Ingenuity Pathway Analysis (IPA) identified crucial pathways and regulatory networks, highlighting IRF5 as a key regulatory factor in high-salt DOCA-induced hypertension or inflammatory conditions. IRF5 appears to influence immune cell, epithelial cell, and cardiomyocyte death and dysfunction through a pro-inflammatory pathway mediated by STAT1, CXCL10, and TNF (Figure 4F). This finding is consistent with our in vivo observations, where high-salt exposure led to fibrosis and apoptosis in the mouse aorta.

Collectively, these findings indicate that under high-salt DOCA-induced hypertension conditions, IRF5 acts as a key regulatory factor, likely interacting with STAT1 and STAT2 within the same regulatory network, contributing to the pathological processes in SSH.

### 2.4. IRF5 Regulates the Phosphorylation and Nuclear Translocation of STAT1 and STAT2 in SSH Mice

**Figure 5:**
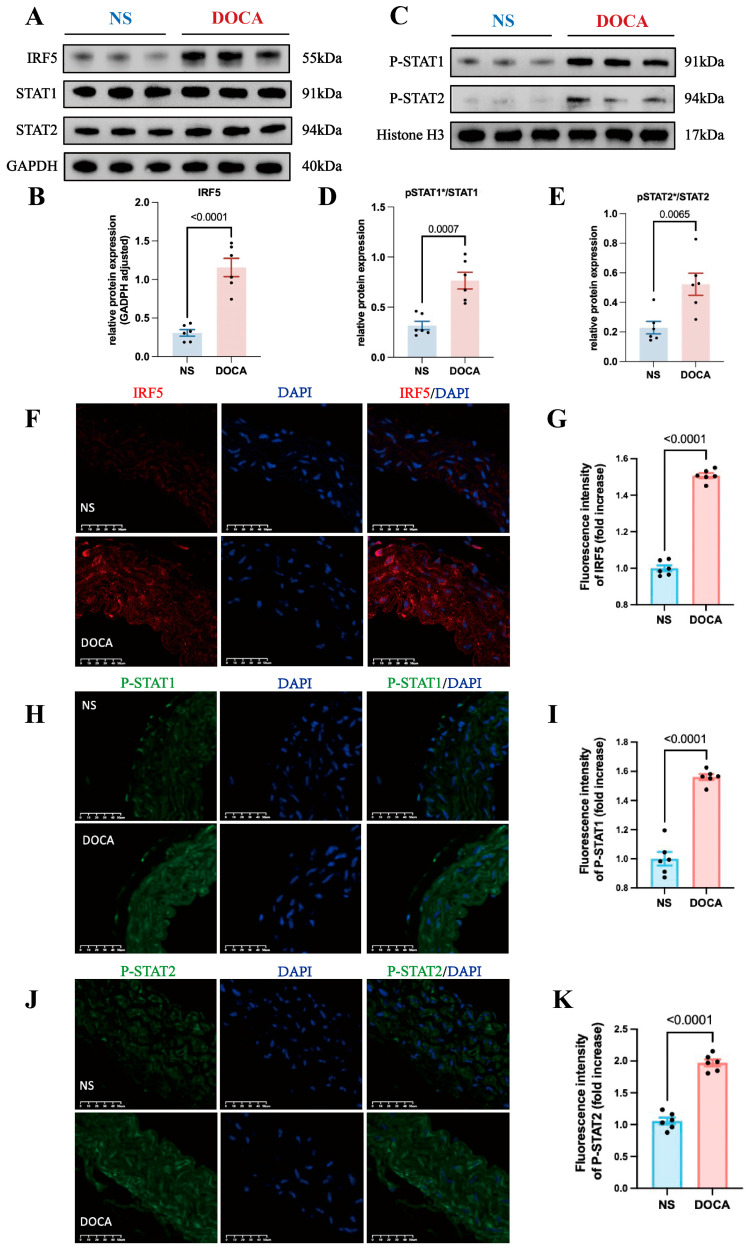
**IRF5 and downstream target expression levels between NS and DOCA groups**. (**A**) Western blot analysis showing the expression levels of IRF5 and total STAT1/2 proteins in aortic tissues. (**B**) IRF5 protein expression level significantly increased in the DOCA group. (**C**) Western blot analysis showing the nuclear expression levels of pSTAT1/2 proteins in aortic tissues. (**D**) pSTAT1/STAT1 ratio and (**E**) pSTAT2/STAT2 ratio significantly increased in the DOCA group. (**F**) Representative images of IRF5 (red) immunofluorescence in aortas of the NS and DOCA groups. Scale bar: 50 μm; blue represents nuclei. (**G**) Fluorescence intensity of IRF5 significantly increased in the abdominal aorta tissue of the DOCA group. (**H**) Representative images of pSTAT1 (green) immunofluorescence in aortas of the NS and DOCA groups. (**I**) pSTAT1 showed higher fluorescence intensity in the DOCA group. (**J**) Representative images of pSTAT2 (green) immunofluorescence in aortas of the NS and DOCA groups. (**K**) pSTAT2 showed higher fluorescence intensity in the DOCA group. * Nuclear protein.

Based on the bioinformatics findings, we hypothesized that IRF5 regulates both STAT1 and STAT2. We extracted total RNA from mice aortic tissues and examined protein expression, finding that IRF5 expression was notably upregulated in the DOCA group (Figure 5A,B). However, there were no significant differences in total STAT1 and STAT2 levels between the DOCA and NS groups (Figure 5A and Figure S3A,B).

Previous research has shown that Type I IFNs’ activation leads to the phosphorylation of STAT2 and then acts as a docking site for latent STAT1 [41]. The phosphorylated STAT1 and STAT2 dimers can translocate from the cytoplasm to the nucleus. Additionally, IRF5 plays a key role in regulating Type I IFN gene transcription. Thus, we hypothesized that IRF5 may influence the phosphorylation and nuclear translocation of STAT1 and STAT2 in SSH. To test this, we extracted nuclear proteins and assessed the expression levels of pSTAT1 and pSTAT2. Western blot analysis revealed that the nuclear levels of both pSTAT1 and pSTAT2 were significantly increased in the DOCA group (Figure 5C and Figure S3C,D), and the ratios of pSTAT1/STAT1 and pSTAT2/STAT2 were also significantly elevated (Figure 5D,E). Immunofluorescence staining of mouse aortic tissue sections revealed a significantly higher expression of IRF5 in the DOCA group compared to the NS group (Figure 5F,G). Additionally, the levels of both pSTAT1 and pSTAT2 were markedly elevated in the DOCA group (Figure 5H–K). These findings further support the role of IRF5 in regulating the phosphorylation and nuclear translocation of STAT1 and STAT2 in SSH.

### 2.5. scRNA-Seq Reveals IRF5 Enrichment in Endothelial Cells of Human and Mouse Aortas 

**Figure 6:**
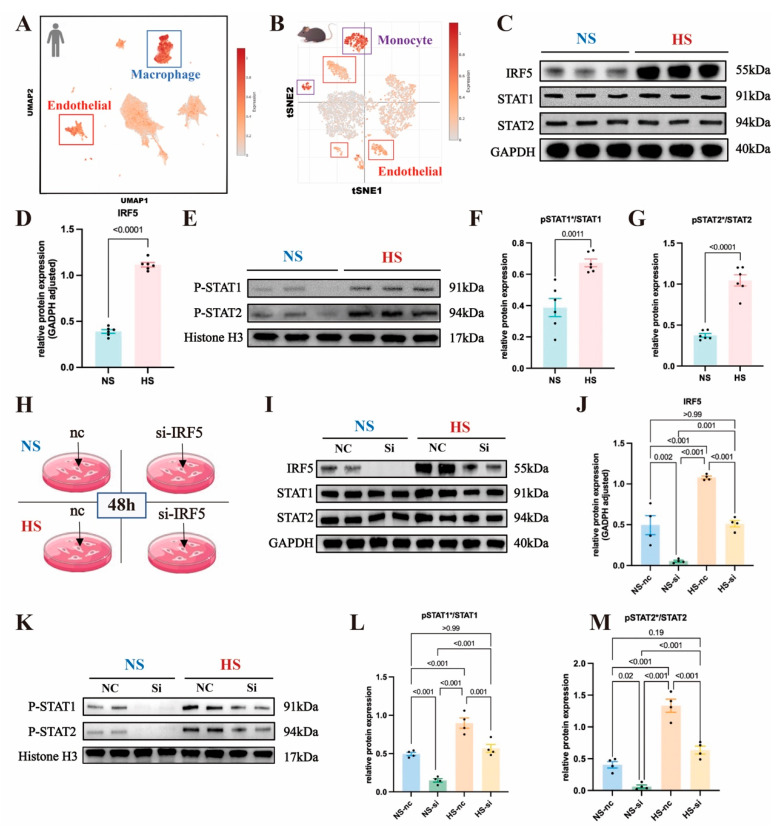
**Analysis of IRF5 and downstream signaling in endothelial cells and cellular populations:** (**A**) Global uniform manifold approximation and projection (UMAP) plot illustrating different cell populations in the normal human aorta using single-cell RNA sequencing data. Each subcluster denotes a distinct cell type, with color gradients indicating the expression levels of IRF5 and downstream transcripts. IRF5 exhibits heightened expression in macrophages and endothelial cells in humans. (**B**) T-Distributed Stochastic Neighbor Embedding (t-SNE) plot depicting diverse cell populations in the normal mouse aorta via single-cell RNA sequencing data. Each subcluster signifies a unique cell type, with color gradients reflecting the expression levels of IRF5 and downstream transcripts. IRF5 demonstrates elevated expression in monocytes and endothelial cells in mice. (**C**) Western blot analysis of IRF5 and total STAT1/2 proteins in primary mouse endothelial cells. (**D**) Increased IRF5 protein expression observed in the DOCA group. (**E**) Western blot analysis showing the nuclear expression levels of pSTAT1/2 proteins in primary mouse endothelial cells. (**F**) Elevated pSTAT1/STAT1 ratio and (**G**) pSTAT2/STAT2 ratio observed in the DOCA group. (**H**) Use of small interfering RNA (siRNA) to silence IRF5 in aortic endothelial cells under normal-salt and high-salt conditions. (**I**) Western blot analysis of IRF5 and total STAT1/2 proteins in mouse primary endothelial cells with or without IRF5 siRNA treatment. (**J**) IRF5 protein expression levels in NS and HS groups with or without siRNA intervention. (**K**) Western blot analysis of pSTAT1/2 proteins in mouse primary endothelial cells with or without IRF5 siRNA treatment. (**L**) pSTAT1/STAT1 ratio and (**M**) pSTAT2/STAT2 ratio in NS and HS groups with or without siRNA intervention. * Nuclear protein.

To determine the expression levels of IRF5 across various cell types within aortic tissue, we conducted analyses using scRNA-seq databases from both human and mouse aortas. For the human aorta, scRNA-seq data from normal aortas were obtained from a previous study and accessed via the single-cell portal [52]. Utilizing uniform manifold approximation and projection (UMAP) plots, we color-coded each subcluster to visualize the distribution and relationships between distinct cell types within the human aorta (Figure S4A). A bidirectional bar graph illustrated the average upregulation and downregulation levels of IRF5 targets across different cell populations (Figure S4B). In Figure 6A, we overlaid IRF5 onto the mean expression levels across various human cellular populations. By comparing the cellular distribution in the UMAP plot, marked with different colors representing subpopulations, we observed an enrichment of IRF5 and its downstream targets in human macrophages and ECs (Figure 6A and Figure S4A). Similarly, for mice samples, scRNA-seq data from normal aortas were sourced from another study [53]. The T-SNE plot, color-coded by subclusters, showed the distinct cell populations within the mice aorta (Figure S4C). In Figure 6B, we displayed the overlay of IRF5 onto the mean expression levels across various mouse cellular populations. Combining the cell position distribution in the UMAP plot represented by different colors, we observed an enrichment of IRF5 and its downstream targets in mouse monocytes and ECs (Figure 6B and Figure S4C).

Based on scRNA-seq analysis, we found that IRF5 is enriched in aortic ECs in both humans and mice. Therefore, we proceeded with in vitro experiments targeting ECs to examine whether IRF5 leads to the phosphorylation and nuclear translocation of STAT1 and STAT2.

### 2.6. IRF5 Induces Phosphorylation and Nuclear Translocation of STAT1 and STAT2 in Aortic ECs

We isolated primary mouse aortic ECs and cultured them in standard ECM (Na^+^ 114 mmol/L) or high-salt ECM (Na^+^ 164 mmol/L). Extraction of aortic EC proteins for Western blot (WB) experiments revealed a significant increase in IRF5 expression in the high-salt (HS) group compared to the normal-salt (NS) group (Figure 6C,D), while the total levels of STAT1 and STAT2 showed no significant differences between the two groups (Figure 6C and Figure S4D,E). Additionally, we isolated nuclear proteins from aortic ECs and measured the expression levels of pSTAT1 and pSTAT2, finding that both pSTAT1 and pSTAT2 were significantly elevated in the HS group (Figure 6E and Figure S4F,G). The ratios of pSTAT1/STAT1 and pSTAT2/STAT2 were also significantly increased (Figure 6F,G).

To investigate the role of IRF5 further, we used siRNA to knock down IRF5 in aortic ECs under both NS and HS conditions and assessed the expression of downstream STAT proteins (Figure 6H). Compared to the negative control group, IRF5 expression levels were significantly reduced in both the NS and HS groups following siRNA intervention (Figure 6I,J). The total amounts of STAT1 and STAT2 remained unaffected by IRF5 siRNA, with no significant differences in their expression levels across the four groups (Figure 6I and Figure S4H,I). We then extracted nuclear proteins to measure the expression levels of pSTAT1 and pSTAT2. Following siRNA intervention, the levels of pSTAT1 and pSTAT2 were significantly decreased in both the NS and HS groups (Figure 6K and Figure S4J,K), and the ratios of pSTAT1/STAT1 and pSTAT2/STAT2 were also significantly reduced (Figure 6L,M). This indicates that IRF5 induces the phosphorylation and nuclear translocation of its downstream targets, STAT1 and STAT2.

Since phosphorylated STAT1 and STAT2 may form the IFN-α-stimulated gene factor 3 complex with IRF9 and translocate to the nucleus, we also assessed the expression level of IRF9. We found that IRF9 expression was not affected by IRF5 siRNA, with no significant differences observed across the four groups (Figure S4L,M). Therefore, we demonstrated that in SSH, IRF5 regulates downstream gene transcription by phosphorylating STAT1 and STAT2, which subsequently translocate to the nucleus.

### 2.7. ESM1 Is a Direct Transcriptional Target of the pSTAT1::pSTAT2 Dimer

Phosphorylated STAT1 and STAT2 translocate to the nucleus, where they possess DNA-binding capabilities, enabling them to bind to target gene promoters and regulate the transcription of downstream genes. We therefore aimed to identify which genes are regulated by the pSTAT1::pSTAT2 dimer.

ESM1 plays a crucial role in endothelial inflammation and serves as a marker of endothelial activation and dysfunction [48]. Our previous research showed that ESM1 levels increased in an L-NAME/high-salt-induced hypertension mouse model and influenced the expression of downstream adhesion molecules [54]. In the present study, RNA-seq analysis revealed a positive correlation between STAT1 and STAT2 transcript levels and ESM1 expression (Figure S5A,B). Based on these transcriptional findings, we further assessed ESM1 expression at the protein level and observed a significant upregulation in the aortic tissue and endothelial cells of DOCA-treated mice (Figure S5C–F). Additionally, siRNA-mediated knockdown of IRF5 in endothelial cells resulted in a marked decrease in ESM1 levels, suggesting that ESM1 functions as a downstream target of IRF5 (Figure S5E,F).

The specific genomic information for the human ESM1 gene is as follows: “>GRCh38.p14 (GCF_000001405.40), chr5, NC_000005.10 (54977867..54985593, complement)”. The promoter sequence of ESM1 can be found in the Supplementary Materials. Using the UCSC database, we predicted transcription factors that could bind to the ESM1 promoter region, setting a threshold of 400. Our analysis revealed that the pSTAT1::pSTAT2 dimer binds to the ESM1 promoter at multiple sites (Figure S5G). Additionally, we utilized the JASPAR database to predict the exact locations and sequences of these binding sites on the ESM1 gene (Table S1). Given these observations, we sought to elucidate the role of pSTAT1 and pSTAT2 in regulating ESM1 transcription following their nuclear translocation and to provide direct evidence supporting their binding to the ESM1 promoter.

**Figure 7:**
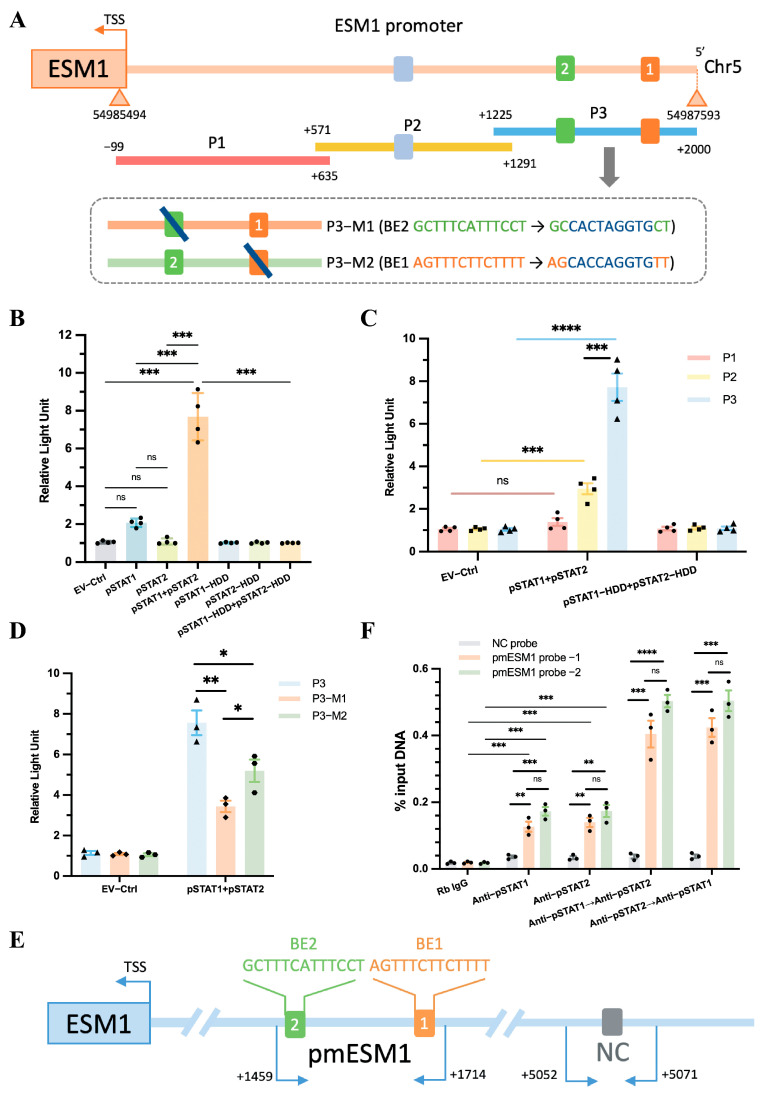
**ESM1 promoter reporters are transactivated by STAT1::STAT2.** (**A**) A diagram shows the relative positions of full-length (FL) and fragments of ESM1 promoter reporters. (**B**) Responses of the FL reporter, and (**C**) the individual fragments of ESM1 promoter to STAT1 and STAT2 or the HDD mutants were investigated. (**D**) Reporter assays of the P3 fragment of the ESM1 promoter containing two mutated Binding elements (BEs) as indicated. (**E**) A schematic illustrates the relative positions of qPCR probes to putative BEs for ChIP-qPCR experiments. (**F**) Antibody-pulled-down chromatins were analyzed by qPCR. Rb, rabbit. TSS, transcription start site. * *p* < 0.05, ** *p* < 0.01, *** *p* < 0.001, **** *p* < 0.0001, ns: not significant.

To determine whether the pSTAT1::pSTAT2 dimer directly interacts with the ESM1 promoter, we cloned the FL ESM1 promoter (~2 kb, Figure 7A) into a luciferase reporter plasmid. This construct was co-transfected into TeloHAEC cells along with various plasmid combinations, including empty vector (EV), pSTAT1, pSTAT2, pSTAT1 + pSTAT2, pSTAT1-HDD, pSTAT2-HDD, or pSTAT1-HDD + pSTAT2-HDD. The results showed that only the pSTAT1 + pSTAT2 co-transfection group exhibited a significant increase in luciferase activity compared to the EV group, while no change in activity was observed in the HDD mutant group (Figure 7B). This finding indicates that pSTAT1 and pSTAT2 function as a dimer and activate ESM1 transcription in a DNA-binding-dependent manner. The upregulation of luciferase activity suggests that the dimer enhances ESM1 transcriptional activity.

Next, the FL reporter was subdivided into three overlapping fragments (P1, P2, and P3, Figure 7A). The P3 fragment contained two predicted pSTAT1::pSTAT2 binding elements (BEs): ‘AGTTTCTTCTTTT’ and ‘GCTTTCATTTCCT’, while P2 contained one predicted BE: ‘TGTTTCCCTTCCC’ (Figure 7A, Supplementary Table S1). Luciferase assays demonstrated that the P3 fragment exhibited a stronger interaction with the pSTAT1::pSTAT2 dimer than the P2 fragment, whereas the P1 fragment showed no interaction with the dimer (Figure 7C). Furthermore, mutating either BE1 or BE2 in the P3 fragment to a DraIII restriction site (CACXXXGTG) still resulted in a positive interaction with the pSTAT1::pSTAT2 dimer, although mutating a single BE reduced pSTAT1::pSTAT2-dependent transactivation (Figure 7A,D). The results indicate that BE1 and BE2 serve as independent binding sites for the pSTAT1::pSTAT2 dimer, and both sites synergistically enhance ESM1 transcriptional activity.

To confirm the direct binding between the P3 fragment and the pSTAT1::pSTAT2 dimer, we performed ChIP followed by quantitative PCR (ChIP-qPCR) to assess the genomic occupancy of the pSTAT1::pSTAT2 dimer at the P3 sequence. We designed three qPCR probe sets: one negative control (NC) probe located 5 kb upstream of the ESM1 transcription start site (TSS) and two probes targeting the putative binding elements within the P3 fragment (Figure 7E, Supplementary Figure S6). Chromatin was immunoprecipitated using anti-pSTAT1, anti-pSTAT2, or sequentially using anti-pSTAT1 followed by re-ChIP with anti-pSTAT2, and vice versa. qPCR analysis showed a preferential enrichment of the pSTAT1::pSTAT2 dimer at the P3 sequence compared to either pSTAT1 or pSTAT2 alone, regardless of the binding order of pSTAT1 and pSTAT2 (Figure 7F).

Luciferase assays combined with ChIP-qPCR results revealed that pSTAT1 and pSTAT2 form dimers that bind to the BE1 and BE2 regions of the ESM1 promoter, enhancing ESM1 transcriptional activity. Although either pSTAT1 or pSTAT2 can independently bind to the ESM1 promoter region (Figure 7F Rb IgG vs. Anti-pSTAT1, Rb IgG vs. Anti-pSTAT2), neither exhibited a significant effect on the transcriptional activity of the ESM1 promoter in isolation (Figure 7B EV-Ctrl vs. pSTAT1, EV-Ctrl vs. pSTAT2), as the specific binding sites of pSTAT1::pSTAT2 to ESM1, BE1 and BE2 may serve as potential therapeutic targets for SSH. Furthermore, as there are currently no publicly available IRF5 inhibitors, we used Connectivity Map (CMap) analysis to identify potential IRF5 inhibitors. The compounds with potential inhibitory effects include JZL-184, AP-26113, and halcinonide, offering new directions for SSH immunotherapy (Supplementary Figure S7).

## 3. Discussion

**Figure 8:**
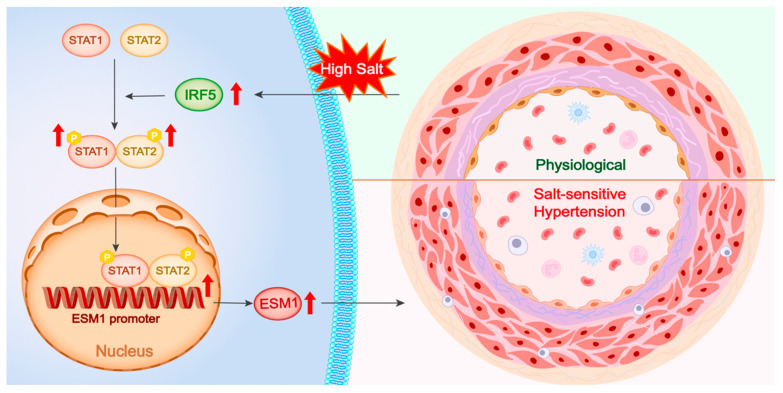
**Central illustration.** High salt stimulation upregulates IRF5 expression, which enhances the phosphorylation and dimerization of STAT1 and STAT2. The activated STAT1/STAT2 complex translocates into the nucleus, binds to the ESM1 promoter region, and promotes ESM1 transcription. Elevated ESM1 expression contributes to vascular remodeling and the development of salt-sensitive hypertension, as illustrated by the transition from a physiological to a pathological vascular phenotype. P, phosphorylation.

This study investigates the regulatory mechanism of the IRF5-STAT1/STAT2-ESM1 pathway in vascular inflammation and endothelial dysfunction in SSH. Using a DOCA-high salt-induced SSH mouse model, we found that a high-salt environment significantly upregulated IRF5 expression in aortic tissue, which in turn drove the phosphorylation and nuclear translocation of STAT1 and STAT2. Single-cell transcriptomic analysis of human and mouse aortas revealed that IRF5 is specifically enriched in vascular ECs, suggesting its critical regulatory role in ECs. Therefore, we selected ECs for in vitro studies and further validated that IRF5 regulates STAT1/STAT2 phosphorylation and dimerization, promoting the nuclear translocation of the pSTAT1::pSTAT2 dimer. This complex directly binds to two distinct binding sequences on the ESM1 promoter, thereby enhancing ESM1 transcriptional activity (Figure 8).

Beyond the traditional view of hypertension as resulting from vascular abnormalities and neurogenic dysfunction, research increasingly highlights the role of immune dysregulation and inflammation in hypertensive pathology, especially in SSH [55]. Hypertensive stimuli activate both innate and adaptive immune pathways, particularly through antigen-presenting cells, T cells, and macrophages [56]. These immune cells infiltrate vascular tissues, promoting inflammation, oxidative stress, and vascular remodeling [19]. The role of immune cell populations, including T cells and macrophages, in the vascular inflammatory response in SSH has been well documented [57,58]. These cells contribute to endothelial dysfunction, vascular remodeling, and fibrosis through the release of pro-inflammatory cytokines and chemokines [58]. High salt intake further stimulates immune responses and promotes the production of reactive oxygen species, exacerbating vascular dysfunction and reducing endothelial nitric oxide levels, ultimately impairing endothelium-dependent vasodilation [19,59]. This not only increases blood pressure but also contributes to vascular complications independent of BP levels by enhancing vascular stiffness and dysfunction [19,59]. High sodium intake also triggers the remodeling of small resistance arteries and elastic arteries, further increasing peripheral resistance and vascular stiffening [28,60]. PP is a key marker of arterial stiffness [61], while aortic diameter measurements serve as indicators of vascular remodeling in hypertension [62,63,64]. Ultrasound-based assessments of aortic diameter are well established in hypertension research and have been correlated with disease progression [65]. In this study, PP showed a gradual increasing trend in the DOCA group compared to the NS group. Histological analysis demonstrated a significant increase in the WT/ID ratio, while ultrasound imaging revealed dilation of the thoracic and abdominal aortic lumen in the DOCA group. These findings indicate the presence of arterial stiffness and vascular remodeling during SSH progression. Therefore, our results align with previous observations, highlighting the critical role of vascular remodeling in the pathological changes of SSH [66,67].

This study integrated RNA-seq data from aortic tissues of DOCA model mice with human and mouse scRNA-seq databases. Through comprehensive bioinformatics analyses, we identified a significant upregulation of the IFN-γ and IFN-α inflammatory pathways in SSH individuals. Based on previous studies, IRF5 plays a role in various inflammatory diseases by regulating the expression of type I IFNs and pro-inflammatory cytokines [29,30,31,32,33] and promotes leukocyte adhesion and VCAM-1 expression through the JAK2-STAT3 pathway [37]. Notably, while the pro-inflammatory role of STAT family members, such as STAT3, in hypertension has been reported [46], the role of STAT1/STAT2 in endothelial inflammation remains unclear. In this study, we identified IRF5, STAT1, and STAT2 as key DEGs in the significantly upregulated IFN-γ/α pathway. Further SCENIC analysis confirmed that IRF5 and STAT1 are critical upstream transcriptional regulators. IPA network analysis revealed that IRF5 regulates downstream genes, particularly STAT1, thereby influencing immune cell responses, pro-inflammatory signaling, cell death, and fibroblast activity. These phenotypes align with the pathological features observed in SSH mice, including aortic inflammatory remodeling, apoptosis, and fibrosis. Thus, we identified IRF5 as a key upstream transcription factor and investigated whether it is upregulated in SSH and regulates downstream STAT proteins.

Western blot and immunofluorescence staining of aortic tissues confirmed a significant increase in IRF5 expression in SSH mice. However, this did not directly affect the total levels of STAT1 and STAT2. Previous studies have shown that STAT proteins exist in an inactive form in the cytoplasm and, upon extracellular signal activation, undergo phosphorylation to form homo- or heterodimers, which then translocate to the nucleus to regulate target gene transcription [39,40,41,42]. Thus, IRF5 may regulate STAT signaling by promoting its phosphorylation and nuclear translocation. In this study, phosphorylated STAT1 and STAT2 levels were elevated in nuclear extracts from DOCA mouse aortic tissues, supporting our hypothesis that IRF5 activates downstream signaling by enhancing STAT1/STAT2 phosphorylation and heterodimer formation rather than altering their total protein levels (Figure 8). This mechanism contrasts with STAT3 regulation in SSH, which typically depends on sustained JAK2-mediated phosphorylation [46], suggesting that IRF5 may activate STAT1/STAT2 through a unique noncanonical pathway.

Endothelial dysfunction plays a central role in cardiovascular disease progression, often exacerbated by inflammatory states and metabolic disorders, forming a vicious cycle that further deteriorates vascular function [68]. High salt intake induces microvascular endothelial inflammation, structural remodeling, and functional impairment, even in normotensive individuals [27]. In this study, scRNA-seq analysis revealed significant upregulation of IRF5, specifically in aortic endothelial cells (ECs) from both humans and mice, rather than in macrophages enriched in humans or monocytes enriched in mice. This cross-species conservation suggests that the regulatory role of IRF5 in ECs may represent a common pathological mechanism in SSH. Moreover, ESM1 is a key biomarker of endothelial activation and dysfunction [47,48,49,50,51]. For the first time, we demonstrated that the pSTAT1::pSTAT2 heterodimer directly binds to two independent sites within the ESM1 promoter (Figure 7A,D; Supplementary Figures S6 and S8; Supplementary Table S1). Site-directed mutagenesis experiments showed that the disruption of either binding element significantly reduced ESM1 promoter activity (Figure 7D,F). This confirms that the cooperative binding of the STAT complex is essential for ESM1 transcriptional activation. This finding complements our previous report that TMEM16 promotes arterial smooth muscle inflammation and SSH by upregulating ESM1 expression [54]. In contrast, the present study elucidates the IRF5-STAT axis as an upstream transcriptional regulator of ESM1 from the endothelial perspective, expanding the regulatory network and providing a theoretical basis for potential SSH therapeutic strategies targeting endothelial dysfunction.

To enhance the translational potential of our findings, it is crucial to validate the IRF5-pSTAT1/pSTAT2-ESM1 pathway in human vascular tissues and in other animal models of hypertension. Such validation will provide insights into the relevance of these molecular changes in clinical settings. A limitation of this study is the lack of in vitro validation experiments using IRF5 inhibitors, as no publicly available IRF5 inhibitors are currently available. To address this, we used GSEA and CMap analysis to predict potential compounds that may inhibit IRF5 gene expression, identifying candidates such as JZL-184, AP-26113, and halcinonide.

In addition, due to post-transcriptional modifications, translation control, and protein degradation mechanisms, transcript levels do not always correlate with functional protein levels. In our study, we explored the association between IRF5, STAT, and ESM1 at the transcriptional level and validated these findings at the protein level using Western blot. However, proteomics approaches or functional analyses may be necessary in future studies to further validate the translational effects of these transcriptomic changes. And we used publicly available scRNA-seq data to identify IRF5-enriched cell populations due to the lack of high-quality single-cell datasets for salt-sensitive hypertensive and sham-operated mouse aortas. While this provided initial insights, study-specific factors may influence transcriptomic profiles. Future studies will include single-cell RNA sequencing of aortic tissues from our experimental groups to validate these findings.

A limitation of the DOCA-salt hypertension model is the variability in hypertension development. Some DOCA-treated mice did not develop hypertension, likely due to partial or complete detachment of the subcutaneously implanted DOCA pellet, as mice tend to lick each other’s neck wounds. Despite careful monitoring, pellet loss was unavoidable, leading to the exclusion of non-hypertensive mice. This selection may introduce bias, but it ensures that observed effects are due to hypertension. Future studies could explore alternative DOCA delivery methods, such as osmotic minipumps, to improve model consistency. Additionally, our study did not include uninephrectomy in the sham group, which was performed to minimize unnecessary surgical interventions. However, it is important to note that uninephrectomy itself can have physiological effects, including activation of the renin–angiotensin system. This potential influence of uninephrectomy on the study outcomes may contribute to variability between groups.

## 4. Materials and Methods

### 4.1. Animals

All experimental procedures were conducted in accordance with the guidelines and approval of the Peking Union Medical College Hospital Institutional Animal Care and Use Committee, as evidenced by the approval letter Code (XHDW-2021-034). Adult male C57B/L6J mice (8 weeks old, weighing 20–22 g) were procured from the Chinese Academy of Medical Sciences. The mice were housed under standard conditions with a 12 h light/dark cycle, room temperature (RT) maintained at 19–22 °C, and humidity at 50 ± 10%. All mice were provided with a standard laboratory rodent diet (0.3% sodium) and allowed to eat ad libitum throughout the study. After a 14-day acclimation period, the mice were randomly divided into two groups: the normal salt (NS) group, which served as the sham operation group, and the high-salt/deoxycorticosterone acetate (DOCA) group. Due to the approximately 60% success rate in establishing the DOCA high-salt model, 15 mice were allocated to the NS group and 25 to the DOCA group.

Mice in the DOCA group underwent unilateral nephrectomy under anesthesia induced by an initial dose of tribromoethanol (250 mg/kg, i.p.), providing a stable anesthesia duration of 30–45 min. Supplemental doses (75–125 mg/kg, i.p.) were administered as needed when signs of recovery, such as toe pinch reflex or spontaneous movement, were observed to maintain adequate anesthesia. The skin over the left flank was shaved, and a 1.5 cm incision was made through the skin and underlying muscle caudal to the rib cage. The left kidney was exteriorized and removed after ligation of the renal artery and vein with 5–0 silk sutures. The muscle and skin layers were then closed separately with 3–0 silk sutures. A small area between the shoulder blades was shaved, and a 1 cm incision was made through which sustained-release DOCA tablets (#M-121, Innovative Research of America, Sarasota, FL, USA) were implanted subcutaneously, providing a dose of 50 mg DOCA per mouse. Mice in the DOCA group were provided with water containing 1.0% NaCl (#S805275, Macklin, Shanghai, China) and 0.2% KCl (#AS744, GPC, Wuhan, China), along with a standard laboratory diet throughout the study. The criteria for successful model establishment were defined as blood pressure levels exceeding 140/90 mmHg at 4 weeks after the implantation of DOCA sustained-release tablets.

For the sham group (NS), the same surgical procedure was performed, including the flank incision and exposure of the left kidney, but without actual nephrectomy or implantation of DOCA tablets. The incision was closed in the same manner as the nephrectomy group. Mice in the NS group were continuously provided with tap water and a standard laboratory diet throughout the study.

The success of the DOCA model was determined based on blood pressure measurements taken using the non-invasive tail-cuff method after four weeks of DOCA pellet implantation. The model was considered successfully established if the systolic blood pressure (SBP) and diastolic blood pressure (DBP) reached or exceeded 140 mmHg and 90 mmHg, respectively. Only those that exhibited a sustained increase in SBP and DBP were included for further analysis. This approach ensures that the hypertensive phenotype is robustly established. In this study, 19 mice in the DOCA group met these criteria, resulting in a final sample size of *n* = 19.

### 4.2. Blood Pressure Measurement

Average SBP and DBP were measured using the non-invasive tail-cuff method (#BP-2010 Series, Softron, Beijing, China). Mice underwent training for three days each week for two weeks prior to measurements. BP was consistently recorded during a fixed time period each day, specifically between 2:00 PM and 5:00 PM. Eight to ten measurements were taken each week for each mouse, and the averages were calculated from these readings.

### 4.3. Ultrasound Imaging

Ultrasound imaging (Vevo 3100, Fujifilm VisualSonics Inc., Toronto, ON, Canada) was performed at the time of DOCA model establishment, specifically 4 weeks after DOCA implantation. Mice were initially anesthetized with tribromoethanol (150 mg/kg, i.p.), and then maintained on 2% isoflurane in 95% oxygen to keep the heart rate at 490 ± 50 bpm throughout the procedure. For the thoracic aorta, the long axis was obtained at the level of the aortic arch, while the short axis was captured near the aortic valve. For the abdominal aorta, both the long and short axes were measured at the level just below the renal arteries. The imaging was conducted using a high-resolution ultrasound system equipped with a 30 MHz transducer to visualize the aortic structure and function accurately. Images were recorded for subsequent analysis of aortic diameter and vascular function.

### 4.4. Aorta Tissue Isolation and RNA Sequencing

Aorta samples were collected four weeks after DOCA implantation, representing a late stage of hypertension in this model, characterized by well-established SBP levels. Mice were anesthetized with trichloroethanol (400 mg/kg, intraperitoneally) and euthanized by cervical dislocation. After euthanasia, mice were perfused with cold phosphate-buffered saline (PBS) to remove blood from the vasculature. The aorta was carefully dissected from the ascending thoracic region to the abdominal bifurcation under a stereomicroscope, avoiding contamination from surrounding tissues. All procedures were performed on ice to maintain tissue integrity. The isolated aortic tissues were immediately placed in RNAlater™ solution (#AM7020, Thermo Fisher Scientific, Waltham, MA, USA) and stored at ™80 °C until further processing. The aortic tissues for sequencing were sent directly to Novogene (Beijing, China) for RNA extraction, library preparation, and RNA sequencing using the Illumina platform (Illumina, Inc., San Diego, CA, USA).

### 4.5. Histological and Apoptosis Assays

Aortic samples from the NS and DOCA groups were fixed in 4% paraformaldehyde for 24 h, dehydrated, embedded in paraffin, and sectioned into 3.5 μm slices. All aortic pathological sections were obtained from the proximal segment of the descending thoracic aorta to minimize anatomical variation-induced errors. Hematoxylin and eosin (H&E) staining (#C0105S, Beyotime, Shanghai, China) and Masson staining (#C0189S, Beyotime, Shanghai, China) were performed following the manufacturer’s instructions. The sections were imaged using a Pannoramic 250 scanner (3DHISTECH, Budapest, Hungary) to capture the entire aortic cross-section. Wall thickness and inner diameter were measured using K-Viewer (v1.0.2), and fibrosis analysis was conducted with ImageJ 1.53 (National Institutes of Health, Bethesda, MD, USA). For each group, four aortic samples were analyzed for H&E staining and seven samples for Masson staining. Terminal deoxynucleotidyl transferase dUTP nick end labeling (TUNEL) staining was performed using the One Step TUNEL Apoptosis Assay Kit (#C1086, Beyotime, Shanghai, China) to detect apoptotic cells. Sections were treated with 2% proteinase K, followed by incubation in the TUNEL reaction mixture, and visualized with 3,3′-Diaminobenzidine. Images for TUNEL staining were captured using the fluorescence microscope (NIKON ECLIPSE TI-SR, Tokyo, Japan). Fluorescence intensity was analyzed using ImageJ, with three aortic samples per group. All histological analyses were conducted in a blinded manner.

### 4.6. Immunofluorescent Staining

All aortic pathological sections were collected from the proximal segment of the descending thoracic aorta to reduce errors caused by anatomical variation. The slides were deparaffinized by sequentially washing with xylene I and xylene II for 15 min each, followed by absolute ethyl alcohol for 15 min. This was followed by washes with 85% ethanol and 75% ethanol for 5 min each, and finally with distilled water, all in a decolorization shaker. For antigen retrieval, the sections were placed in citrate solution and heated at 37 °C for 10 min. After retrieval, the slides were rinsed three times with PBS (pH 7.4) for 5 min each in the shaker and then incubated overnight at 4 °C with primary antibodies: rabbit monoclonal IRF5 (#ab181553, 1:200, Abcam, Cambridge, UK), mouse monoclonal STAT1 (#sc-464, 1:100, Santa Cruz Biotechnology, Inc., Dallas, TX, USA), and mouse monoclonal STAT2 (#sc-166201, 1:100, Santa Cruz Biotechnology, Inc., Dallas, TX, USA). Following incubation with primary antibodies, the sections were rinsed three times with PBS for 5 min each. The slides were then incubated for 50 min at RT with secondary antibodies: goat anti-rabbit (#111-165-003, 1:200, Jackson ImmunoResearch, West Grove, PA, USA) and goat anti-mouse (#115-165-003, 1:200, Jackson ImmunoResearch, West Grove, PA, USA). The sections were subsequently counterstained with 4′,6-diamidino-2-phenylindole (DAPI, #C0060, 1:100, Solarbio, Beijing, China) for 10 min in the dark. Finally, images were captured using the previously mentioned NIKON fluorescence microscope. For each group, three aortic samples were analyzed, with two locations measured per sample to assess fluorescence intensity. Quantitative analysis of the immunofluorescence was performed using ImageJ. All immunostaining analyses were conducted in a blinded manner.

### 4.7. Western Blot Analysis

Total proteins from aortic tissue and endothelial cells (ECs) were extracted using radioimmunoprecipitation assay buffer (RIPA, #R0020, Solarbio, Beijing, China), while nuclear proteins were isolated using the Protein Extraction Kit (#P0027, Beyotime, Shanghai, China) according to the manufacturer’s instructions. Protease Inhibitor Cocktail (#5871, Cell Signaling Technology, Danvers, MA, USA) and Phosphatase Inhibitor Cocktail (#5870, Cell Signaling Technology, Danvers, MA, USA) were used in all procedures of protein extraction. Protein concentrations were determined with the Pierce BCA Protein Assay Kit (#23227, Invitrogen, Waltham, MA, USA). Optical density was measured at 562 nm using a microplate reader (Synergy H1, BioTek, Winooski, VT, USA).

The extracted proteins were separated by 10% sodium dodecyl sulfate–polyacrylamide gel electrophoresis (SDS-PAGE, #PG01010-S, Solarbio, Beijing, China; #ET15010Gel, ACE, Wuhan, China) and subsequently transferred onto 0.45 μm polyvinylidene fluoride membranes (PVDF, #IPVH00010, Millipore, Billerica, MA, USA). The membranes were blocked and then incubated overnight at 4°C with the following primary antibodies: IRF5 (#ab181553, 1:1000, Abcam, Cambridge, UK), STAT1 (#sc-464, 1:1000, Santa Cruz Biotechnology, Inc., Dallas, TX, USA), Phosphorylated STAT1 (pSTAT1, #ab109461, 1:1000, Abcam, Cambridge, UK), STAT2 (#sc-166201, 1:1000, Santa Cruz Biotechnology, Inc., Dallas, TX, USA), Phosphorylated STAT2 (pSTAT2, #bs-3428R, 1:1000, Bioss, Beijing, China), Histone H3 (#ab1791, 1:1000, Abcam, Cambridge, UK), and Glyceraldehyde-3-phosphate dehydrogenase (GAPDH, #MA5-15738, 1:2000, Invitrogen, Waltham, MA, USA). After washing, membranes were incubated for 1 h at RT with the appropriate secondary IgG antibodies (Anti-Mouse: #ab6789, 1:5000, Abcam; Anti-Rabbit: #ab6721, 1:5000, Abcam, Cambridge, UK). Horseradish Peroxidase detection was performed using Enhanced chemiluminescence reagents (#WBKLS0100, Millipore, Billerica, MA, USA; #PE0010, Solarbio, Beijing, China), and signals were visualized using a BioImaging System (UVP Inc., Upland, CA, USA). Quantitative analysis of protein expression was conducted using ImageJ 1.53.

### 4.8. Isolation and Culture of Endothelial Cells

After the establishment of the DOCA model, mice in both the NS group and the DOCA group were euthanized. The aortas were carefully excised and placed in cold PBS to minimize tissue degradation. To isolate the ECs, the aorta was flushed with PBS and subsequently treated with collagenase type I (1 mg/mL, #17100017, Gibco, Waltham, MA, USA) for 30 min at 37 °C, facilitating the dissociation of ECs from the underlying smooth muscle and extracellular matrix. Following digestion, the aorta was gently agitated to promote cell release, and the resulting cell suspension was passed through a 70 µm cell strainer (BD Falcon, Franklin Lakes, NJ, USA) to eliminate any tissue debris. The isolated cells were then centrifuged at 300× *g* for 5 min, and the pellet was resuspended in the appropriate culture medium. After endothelial cell isolation and culture, microscopic observation confirmed the cells exhibited typical endothelial morphology, including a flat or polygonal shape and tight junctions. No contamination or atypical cells were observed, indicating high purity of the culture.

ECs from the NS group were cultured in a standard endothelial cell culture medium (ECM) (#1001, Sciencell, Carlsbad, CA, USA), mimicking the physiological Na^+^ concentration of approximately 114 mmol/L. In contrast, ECs from the DOCA group were cultured in ECM supplemented with an additional 50 mM NaCl to mimic a high-salt environment (Na^+^ 164 mmol/L). This concentration was determined through a salt concentration gradient assay, which indicated a significant increase in IRF5 protein expression, while the Cell Counting Kit-8 assay demonstrated that cell apoptosis remained appropriately at 50%. The cells were incubated at 37 °C in a humidified atmosphere with 5% CO_2_ for 48–72 h until they reached approximately 80% confluency.

### 4.9. IRF5 Small Interfering RNA (siRNA) Transfection

In a six-well tissue culture plate, 2 × 10^5^ cells were seeded per well in 2 mL of antibiotic-free standard ECM (Na^+^ 114 mmol/L) or high-salt ECM (Na^+^ 164 mmol/L). The cells were then incubated at 37 °C in a CO_2_ incubator for 18–24 h until they reached 60–80% confluency. Subsequently, IRF5 siRNA transfection was performed according to the manufacturer’s instructions (#sc-72045, Santa Cruz Biotechnology, Dallas, TX, USA). To prepare the transfection mixture, the siRNA duplex solution (0.6 pmol/µL) was added to the diluted Transfection Reagent, and the solution was mixed gently before being incubated at RT for 15–45 min. Then, wash the cells with 2 mL of siRNA Transfection Medium (#sc-36868, Santa Cruz Biotechnology, Dallas, TX, USA) and proceed immediately to the next step. Add 0.8 mL of siRNA Transfection Medium to the tube containing the siRNA Transfection Reagent mixture, mix gently, and overlay it onto the washed cells. Incubate the cells as well as fluorescein Conjugated Control siRNA for 6 h at 37 °C in a CO_2_ incubator. After incubation, add 1 mL standard ECM or high-salt ECM with double serum and antibiotic concentration without removing the transfection mixture. Incubate for an additional 18–24 h, then replace the medium with fresh standard ECM or high-salt ECM. Assay the cells 24–72 h after adding the fresh ECM.

### 4.10. Luciferase Reporter Assay

Human Aortic Endothelial Cells (TeloHAEC, #CRL-4052, ATCC, Manassas, VA, USA) were cultured in Vascular Cell Basal Medium (#PCS-100-030, ATCC, Manassas, VA, USA) supplemented with an additional 50 mM NaCl to simulate a high-salt environment, as determined by a salt concentration gradient assay that showed a significant increase in IRF5 protein expression while maintaining cell apoptosis at an appropriate 50%. TeloHAEC (1 × 10^5^) were seeded in a 24-well plate and transfected at 80% confluency using the K-2 transfection reagent (#T060, Biontex, Martinsried, Germany). The human ESM1 promoter’s full-length (FL) fragment (−1180 to +37: NC_000005) was cloned upstream of a luciferase reporter gene in the pGL4.17 vector (#E6741, Promega, Madison, WI, USA). To assess the impact of pSTAT1 and pSTAT2 on the ESM1 promoter, TeloHAEC were co-transfected with the pGL4.17-ESM1 promoter vector along with either pSTAT1 or pSTAT2 expression vectors, pSTAT1 and pSTAT2 expression vectors, or the homeodomain deletion mutant (HDD) of pSTAT1 or pSTAT2, which lacks DNA-binding activity. Negative controls included cells transfected with an empty vector instead of the pSTAT1 and pSTAT2 expression vectors. Additionally, cells were co-transfected with the pCMV-SPORT-βgal plasmid (#10586-014, Life Technologies, Carlsbad, CA, USA) for normalization purposes. To precisely identify pSTAT1 and pSTAT2 dimer binding sites on the ESM1 promoter, the FL reporter was divided into three overlapping segments (P1, P2, and P3, as shown in Supplementary Material), and each segment was individually cloned into the vector, replacing the FL fragment. Luciferase activity was quantified using the PicaGene luciferase assay system (Toyo Ink Group, Tokyo, Japan) and normalized to β-galactosidase activity.

### 4.11. Chromatin Immunoprecipitation (ChIP), re-ChIP, and Quantitative Reverse Transcription Polymerase Chain Reaction (qRT-PCR)

ChIP and re-ChIP were performed to investigate the binding of pSTAT1 and pSTAT2 to the ESM1 gene promoter region. ChIP was conducted using the SimpleChIP^®^ Plus Enzymatic Chromatin IP Kit (#9005S, Cell Signaling Technology, Danvers, MA, USA), following the manufacturer’s protocol with slight modifications. Briefly, cells were transfected with an IRF5 expression vector using Lipofectamine 3000 (#L3000015, Invitrogen, Waltham, MA, USA) to induce STAT1 and STAT2 phosphorylation. After 48 h, the cells were cross-linked with 1% formaldehyde in PBS for 10 min at RT, followed by quenching the reaction with 2.5 M glycine for 5 min at RT and washed with PBS three times. After nuclei were prepared, chromatin was digested using micrococcal nuclease for 20 min at 37 °C, and sonication was performed to ensure the chromatin fragments were between 150 and 900 bp. The chromatin supernatant samples were immunoprecipitated by being incubated with 3 μL of anti-pSTAT1 (#9167, Cell Signaling Technology, Danvers, MA, USA) antibody, anti-pSTAT2 (#ab191601, Abcam, Cambridge, UK), or normal rabbit IgG (#2729, Cell Signaling Technology, Danvers, MA, USA), at 4 °C overnight (12–16 h). The next day, 30 µL of Protein G Magnetic Beads was added and incubated for 2 h at 4 °C with rotation. After incubation, the beads were washed three times with low-salt buffer and once with high-salt buffer at 4 °C for 5 min with rotation per wash. Chromatin was eluted using ChIP elution buffer at 65 °C with gentle vortex mixing (1200 rpm) for 30 min. Crosslinks were reversed by treatment with 5 M NaCl and proteinase K overnight at 65 °C. Samples were then treated with RNase A at 37 °C for 1 h. CHIP DNA was purified and subsequently quantified by qRT-PCR using primers targeting the P3 segment of the ESM1 gene promoter (Figure S6). Gene expression levels were normalized to the expression level of beta-Actin Data analysis was finally presented as percentages of the input DNA. Re-ChIP assays were conducted using the Re-ChIP-IT kit (#53016, Active Motif, Carlsbad, CA, USA). In brief, the chromatin precipitated from the initial ChIP reaction was eluted with 100 µL of diluted Re-ChIP-IT elution buffer at RT for 30 min. The eluted chromatin was then desalted using the desalting column included in the kit. The second ChIP was performed by combining 30 µL of Protein G magnetic beads, 90 µL of the desalted chromatin, and 3 µL of the second CHIP antibody. After washing, the second precipitate was eluted and reverse cross-linked using the same procedure as the first ChIP. DNA was purified by phenol and phenol/chloroform extractions, and subjected to qRT-PCR evaluation.

### 4.12. Statistics and Reproducibility

All immunoassays and staining experiments were performed at least three times, and the results were similar across repetitions. Differences between groups were assessed using one-way analysis of variance followed by the Newman–Keuls post hoc test or unpaired *t*-tests as appropriate. Data are presented as mean ± standard error of the mean. A *p*-value of <0.05 was considered statistically significant. Statistical analyses were conducted using GraphPad Prism version 10.1.1 (GraphPad Software Inc., San Diego, CA, USA). All computed *p*-values were derived from at least three independent experiments, each consisting of three technical replicates.

## 5. Conclusions

Our study showed that IRF5 mediates the phosphorylation of STAT1 and STAT2 in SSH, which increases ESM1 promoter activity. These findings emphasize the important role of IRF5 in regulating arterial inflammation in SSH. They also suggest that IRF5 inhibitors and specific binding sites in the ESM1 promoter may serve as new targets for immune-based therapies for hypertension.

## Data Availability

The datasets used and/or analysed during the current study are available from the corresponding author on reasonable request.

## Supplementary Materials

The following supporting information can be downloaded at https://www.mdpi.com/article/10.3390/ijms26083722/s1.

## Abbreviations

Binding elements (BEs), blood pressure (BP), Chromatin Immunoprecipitation (ChIP), ChIP followed by quantitative PCR (ChIP-qPCR), Connectivity Map (CMap), Diastolic blood pressure (DBP), Deoxycorticosterone acetate (DOCA), Differentially expressed genes (DEGs), Endothelial cell culture medium (ECM), Endothelial cells (ECs), Endothelial cell-specific molecule 1 (ESM1), Empty vector (EV), FL (Full-length), Gene Ontology (GO), Gene Set Enrichment Analysis (GSEA), Glyceraldehyde-3-phosphate dehydrogenase (GAPDH), Homeodomain deletion mutant (HDD), Hematoxylin and eosin (H&E), High-salt (HS), Human Aortic Endothelial Cells (TeloHAEC), Intercellular adhesion molecule-1 (ICAM1), Interferon (IFN), Interferon regulatory factor (IRF), Ingenuity Pathway Analysis (IPA), Janus kinase 2 (JAK2), Negative control (NC), normal salt (NS), Phosphate-buffered saline (PBS), pulse pressure (PP), Phosphorylated STAT1 (pSTAT1), Phosphorylated STAT2 (pSTAT2), Quantitative Reverse Transcription Polymerase Chain Reaction (qRT-PCR), Room temperature (RT), RNA sequencing (RNA-Seq), Salt-sensitive hypertension (SSH), Single-cell RNA sequencing (scRNA-seq), Single-cell Regulatory Network Inference and Clustering (SCENIC), Signal transducer and activator of transcription (STAT), Small Interfering RNA (siRNA), Systolic blood pressure (SBP), Transcription start site (TSS), Terminal deoxynucleotidyl transferase dUTP nick end labeling (TUNEL), Uniform manifold approximation and projection (UMAP), Vascular cell adhesion molecule-1 (VCAM-1), Wall thickness to inner diameter ratio (WT/ID).
