# Supplementary material for: IRF5 Mediates Artery Inflammation in Salt-Sensitive Hypertension by Regulating STAT1 and STAT2 Phosphorylation to Increase ESM1 Transcription: Insights from Bioinformatics and Mechanistic Analysis
Source: Int J Mol Sci. 2025 Apr 15;26(8):3722. doi: 10.3390/ijms26083722 (PMC12027925; doi:10.3390/ijms26083722)
Supplement: Supplementary file 1 [file ijms-26-03722-s001.zip › ijms-3505549-supplementary.pdf]

A

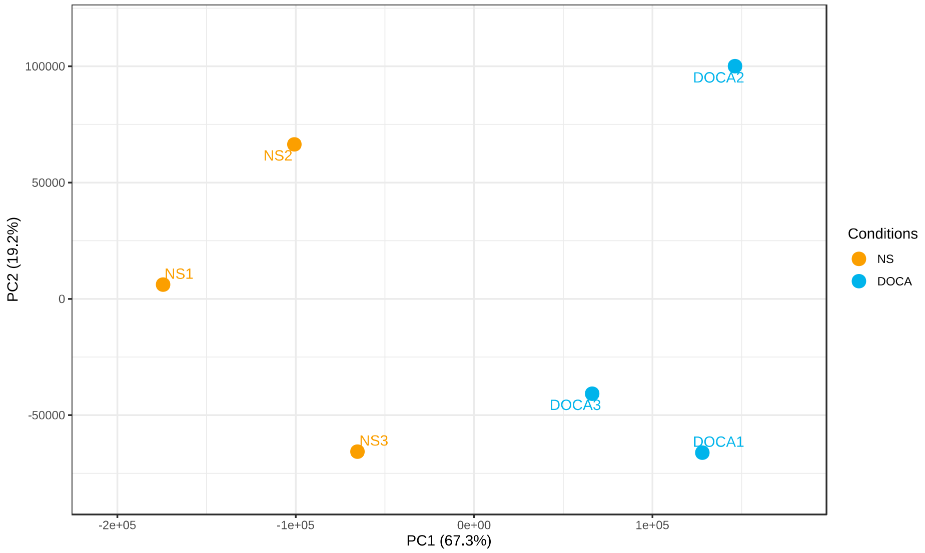

B

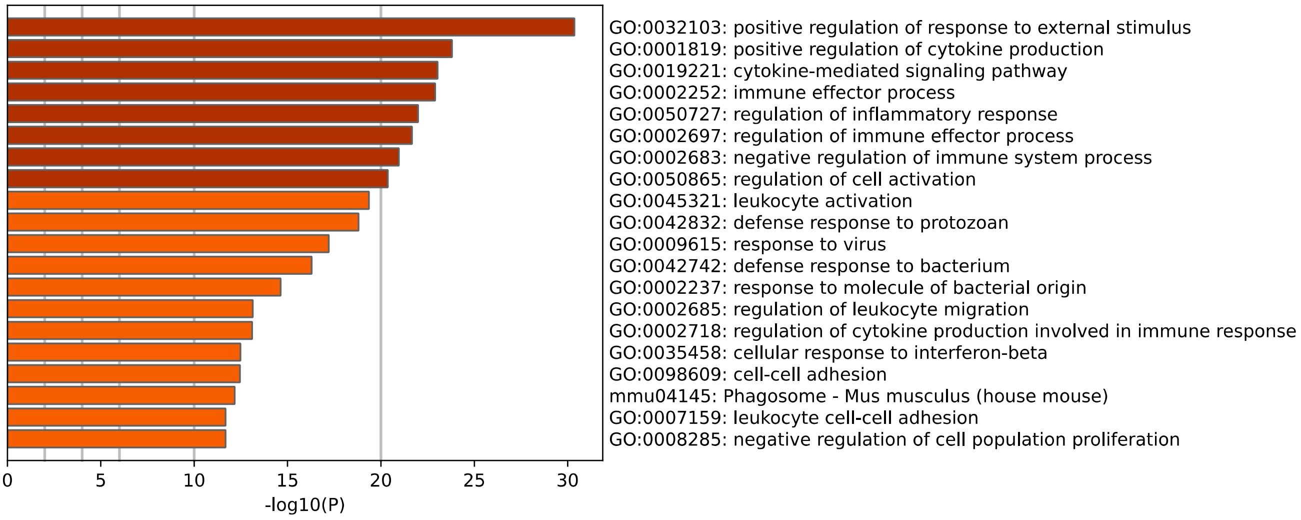

Supplementary Figure S1.

(A) Principal Component Analysis (PCA) of the DOCA group and NS group. (B) Gene Ontology (GO) term enrichment analysis of differentially expressed genes (DEGs).

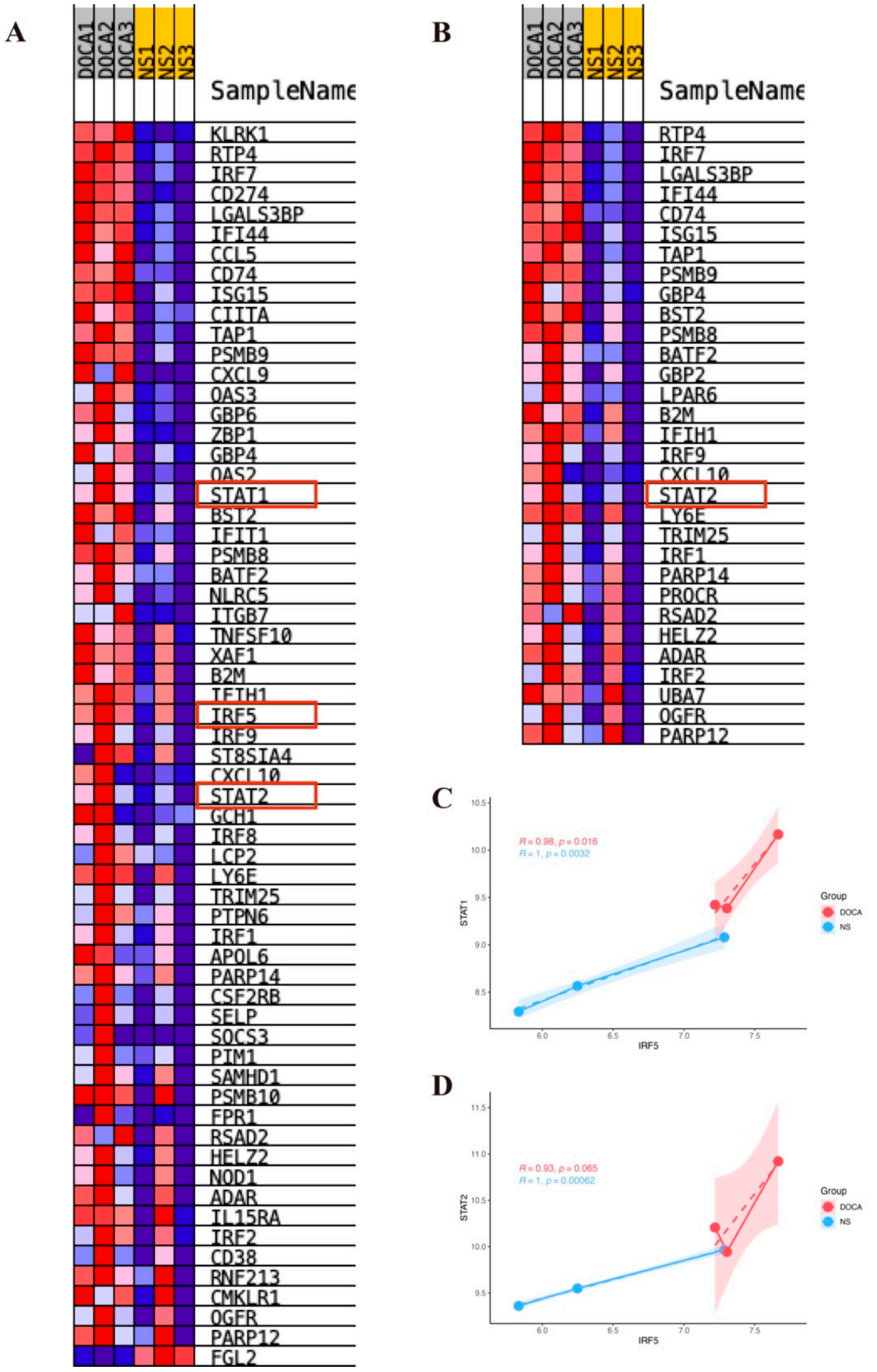

Supplementary Figure S2.

(A) Differentially expressed genes enriched in the Interferon-γ pathway. (B) Differentially expressed genes enriched in the Interferon-α pathway. (C) Pearson linear regression analysis of IRF5 and STAT1 in RNA-seq data. (D) Pearson linear regression analysis of IRF5 and STAT2 in RNA-seq data.

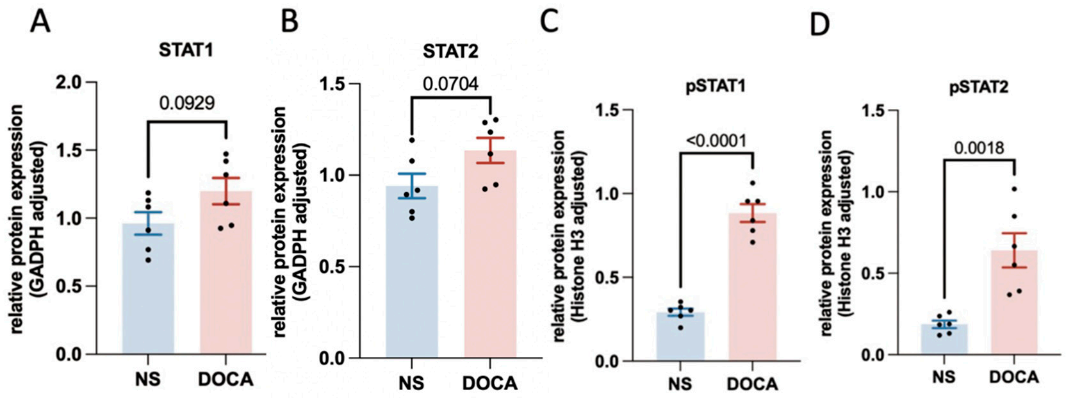

Supplementary Figure S3.

(A) Total STAT1 protein expression levels showed no difference between NS and DOCA groups. (B) Total STAT2 protein expression levels showed no difference between NS and DOCA groups. (C) pSTAT1 protein expression levels showed a significant increase in the DOCA group. (D) pSTAT2 protein expression levels showed a significant increase in the DOCA group.

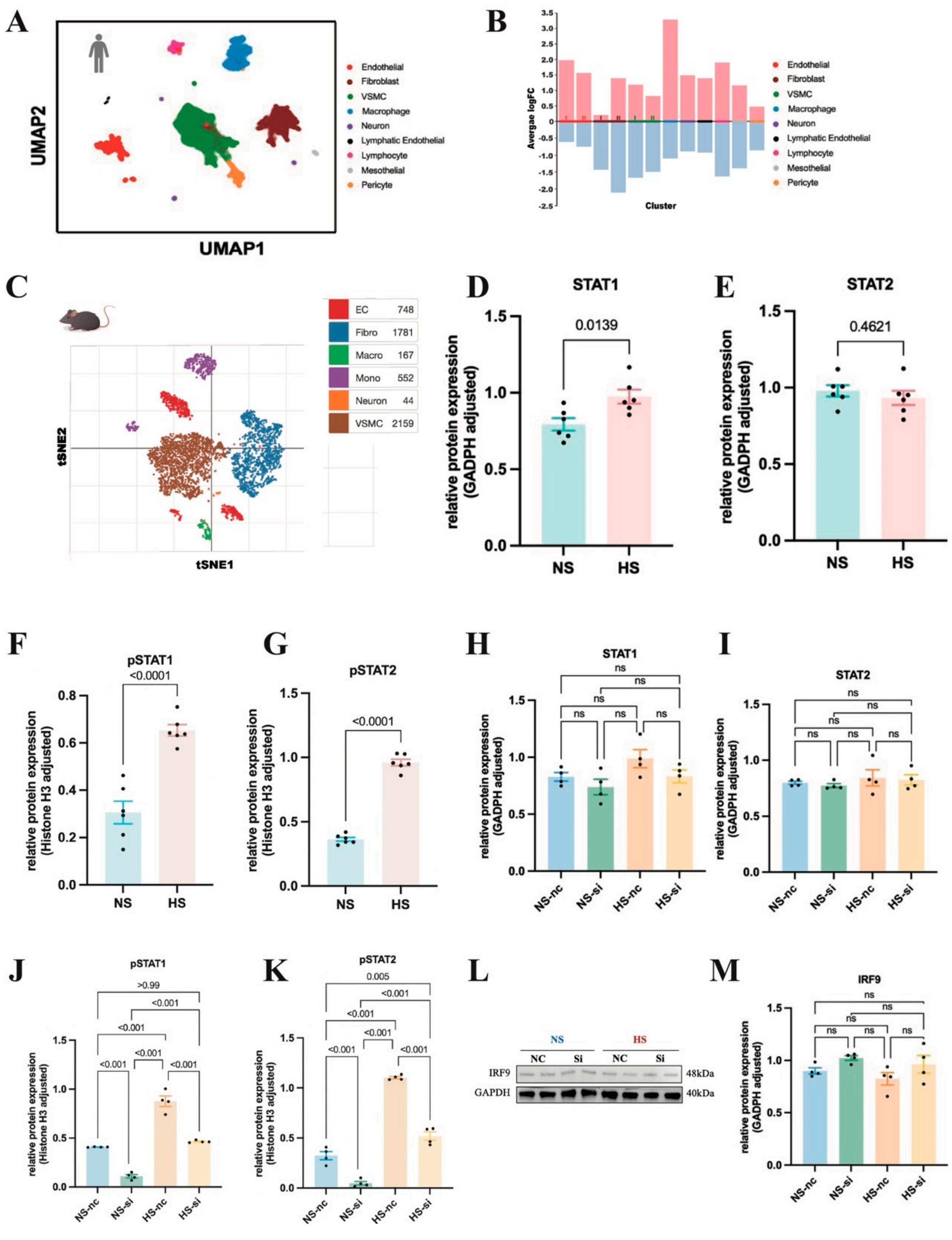

Supplementary Figure S4.

(A) Global uniform manifold approximation and projection (UMAP) plot of the different cell populations in normal human aorta using single-cell RNA sequencing data. Each dot is colored according to its respective subcluster. (B) Average upregulated and downregulated expression levels of IRF5 targets across different cell types. (C) T-Distributed Stochastic Neighbor Embedding (t-SNE) plot of the different cell populations in normal mouse aorta using single-cell RNA sequencing data. Each

dot represents an individual cell and is colored by its respective subcluster. (D) Total STAT1 and (E) Total STAT2 protein expression levels showed no difference between NS and HS groups. (F) pSTAT1 and (G) pSTAT2 nuclear protein expression levels showed a significant increase in the HS group. (H) Total STAT1, (I) Total STAT2, (J) pSTAT1, and (K) pSTAT2 protein expression levels between NS and HS groups with or without IRF5 siRNA intervention. (L) and (M) IRF9 protein expression levels showed no difference between NS and HS groups.

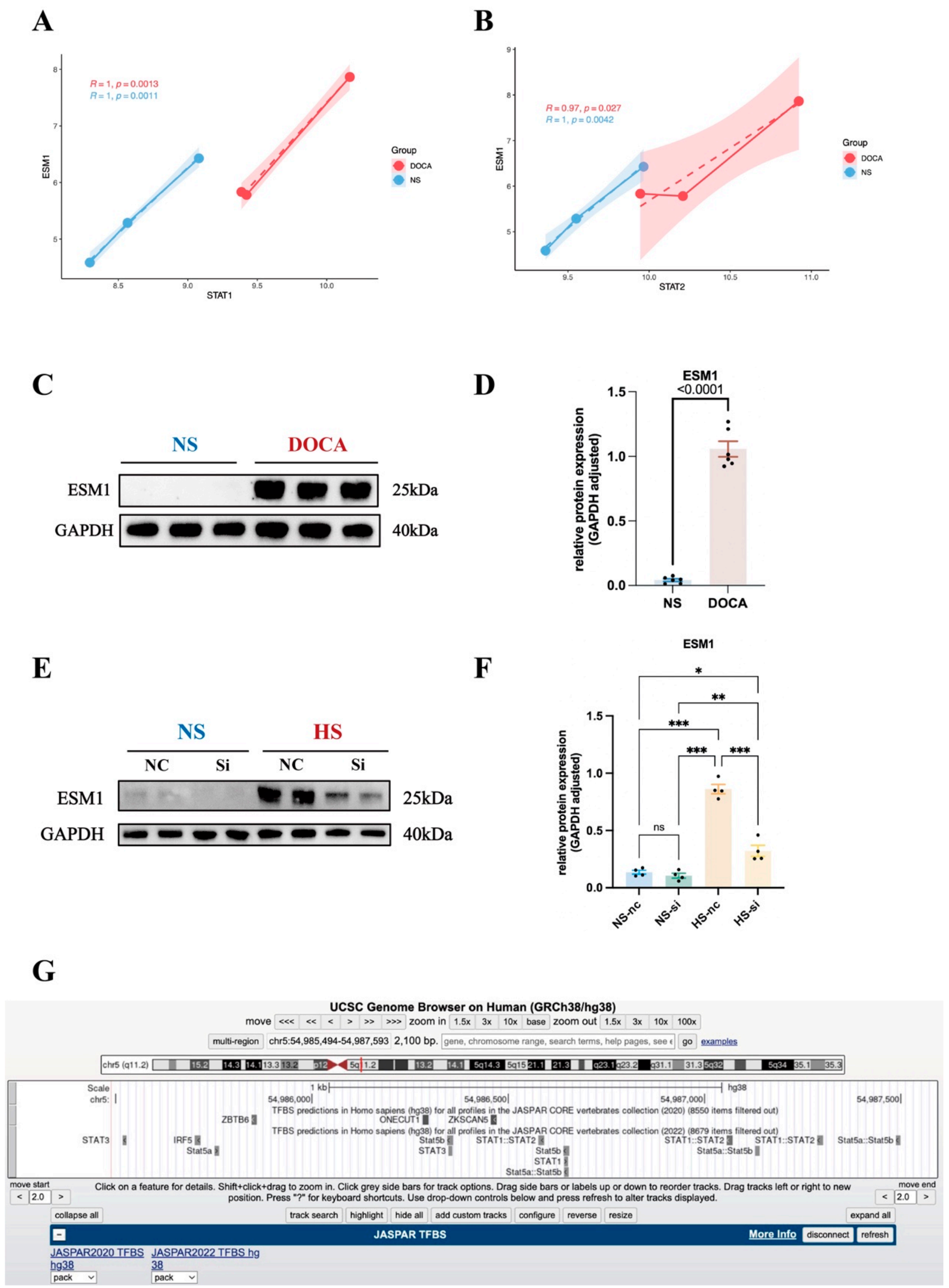

Supplementary Figure S5.

(A) Pearson linear regression analysis of ESM1 and STAT1 in RNA-seq data. (B) Pearson linear regression analysis of ESM1 and STAT2 in RNA-seq data. (C) Western blot analysis showing the expression levels of ESM1 proteins in aortic tissues. (D) ESM1 protein expression level significantly increased in the DOCA group. (E) Western blot analysis of ESM1 proteins in mouse primary endothelial cells with or

without IRF5 siRNA treatment. (F) ESM1 protein expression levels in NS and HS groups with or without siRNA intervention. (G) Prediction of STAT or IRF Transcription Factor Family Binding to the ESM1 Promoter Region Using UCSC Database, and candidates were screened based on a score threshold of 400.

Matrix ID	Name	Score	Relative score	Sequence ID	Start	End	Strand	Predicted sequence
MA0517.2	MA0517.2.STAT1::STAT2	14.749166	0.9189583897264427	NC_000005.10:c54987593-54985494	525	537	+	GCTTTCATTTCCT
MA0137.4	MA0137.4.STAT1	9.324966	0.9088668509624608	NC_000005.10:c54987593-54985494	942	950	-	CTCTGGGAA
MA0137.4	MA0137.4.STAT1	8.780946	0.9009178902629211	NC_000005.10:c54987593-54985494	1237	1245	-	TTCTGGAAA
MA0137.4	MA0137.4.STAT1	7.8289943	0.8870084457360221	NC_000005.10:c54987593-54985494	454	462	+	TTCTGGGGA
MA0137.4	MA0137.4.STAT1	7.66413	0.884599533587249	NC_000005.10:c54987593-54985494	1832	1840	-	ATCCAGGAA
MA0517.2	MA0517.2.STAT1::STAT2	12.477021	0.884533519000057	NC_000005.10:c54987593-54985494	1005	1017	+	TGTTTCCCTTCCC
MA0137.4	MA0137.4.STAT1	7.53407	0.882699158333664	NC_000005.10:c54987593-54985494	1940	1948	+	TGACGGGAA
MA0137.4	MA0137.4.STAT1	6.2016006	0.8632297735896409	NC_000005.10:c54987593-54985494	645	653	+	CTATGGGAA
MA0517.2	MA0517.2.STAT1::STAT2	10.782948	0.8588669123275287	NC_000005.10:c54987593-54985494	294	306	+	AGTTTCTTCTTTT
MA0137.4	MA0137.4.STAT1	5.0358777	0.8461968079291647	NC_000005.10:c54987593-54985494	1318	1326	-	CTCTGAGAA
MA0137.4	MA0137.4.STAT1	4.935307	0.8447273189152325	NC_000005.10:c54987593-54985494	1832	1840	+	TTCCTGGAT
MA0137.4	MA0137.4.STAT1	4.121454	0.832835697564786	NC_000005.10:c54987593-54985494	300	308	-	TTAAAAGAA
MA0137.4	MA0137.4.STAT1	4.121454	0.832835697564786	NC_000005.10:c54987593-54985494	1308	1316	+	TTAAAAGAA
MA0137.4	MA0137.4.STAT1	3.8186038	0.8284106030407606	NC_000005.10:c54987593-54985494	1340	1348	-	TTCCAGTAC
MA0517.2	MA0517.2.STAT1::STAT2	8.744459	0.8279821082585413	NC_000005.10:c54987593-54985494	509	521	-	AGTTTTAGCTTTC
MA0137.4	MA0137.4.STAT1	3.5561745	0.8245761168248331	NC_000005.10:c54987593-54985494	413	421	-	TTTCAGTAA
MA0137.4	MA0137.4.STAT1	3.5399053	0.8243383992190717	NC_000005.10:c54987593-54985494	98	106	-	TTCTGAGGA
MA0137.4	MA0137.4.STAT1	3.4413824	0.82289883131238	NC_000005.10:c54987593-54985494	413	421	+	TTACTGAAA
MA0137.4	MA0137.4.STAT1	3.4065976	0.8223905731095401	NC_000005.10:c54987593-54985494	84	92	+	TTCCAGTTA
MA0137.4	MA0137.4.STAT1	3.0135782	0.8166479674764001	NC_000005.10:c54987593-54985494	692	700	-	TTTTGAGAA
MA0137.4	MA0137.4.STAT1	2.9682634	0.8159858501953918	NC_000005.10:c54987593-54985494	2023	2031	-	TGGTGGGAA
MA0137.4	MA0137.4.STAT1	2.950433	0.8157253216209249	NC_000005.10:c54987593-54985494	1478	1486	+	TTCTAAGTA
MA0137.4	MA0137.4.STAT1	2.8271964	0.8139246485549317	NC_000005.10:c54987593-54985494	1122	1130	+	TGCCAGGAG
MA1420.1	MA1420.1.IRF5	10.28213	0.8101651255478457	NC_000005.10:c54987593-54985494	1878	1891	+	CATAAACCAAAACA
MA0137.4	MA0137.4.STAT1	2.3520784	0.8069824604798455	NC_000005.10:c54987593-54985494	2066	2074	+	AGCTGGGAA
MA0517.2	MA0517.2.STAT1::STAT2	6.9675136	0.8010599089147291	NC_000005.10:c54987593-54985494	502	514	-	GCTTTCAAATTTT

Supplementary Table S1. Predicted Binding Sites of STAT1, STAT2, and IRF5 in the ESM1 Promoter Region Using JASPAR

Green: P3-BE2

Blue: P2-BE

Orange: P3-BE1

BE: binding site

The P3 fragment sequence of ESM1 promoter

5’TGGTAATCTACATAAACTGCTTAGCATGGCATCTGGCACACAGTCAGATCTCTGTAAAGGTTAGATATTA
TTATTAACCACTATTCCAGTTAACTACTCCTCAGAAAGTGGTGTTCAGTAACAAGGGACATTTTCTAGGG
TCAGCCTAAATTGCGACCTAGGCCATTGGAACTATTGGCCATGAAAGCACAATTATGTATTGTAATAGGC
AATAGAATATGTGGCTCTGACATTAGACTAACATCCATCCCTTCAATATAGGGACCTTAGGCAAATTAACC
TCTTTCTTACTCAGTTTCTTCTTTTAAAAAATAAGCATGATAATAGCAGCCACTTCATAATATTGTGATGAG
GTGCAAATGAGTTAATGCATGTAAAGTGTTTAAGCTATTAACTCAACAAATATTACCCATTACTGAAAACA
TCAGAGTGTGCCACCTGCTAAGTAAGTGCTTCTGGGGAGATAGAGTATTTCAAATTGGATTGTCATGAA
AAATTTGGAAAATTTGAAAGCTAAAACTCTTGCTTTCATTTCCTACTGCCTCAATTCTCTGTATTCTGAATT
TACTCCCTGTGTCCCCCTAAAATTGCATTTGCAAAAGCCGATCTTCAAGTTACATCCAATGCCCGCTCTGC
CTCATCTTCTATGGGAAACAAGAATTTTAGAGGTCAGGTAGCCTAACACCATCAATTCTCAAAAGAGGA
AGCTGAGGCCAAGAGAAGTCCTGTGAATT

Orange and Green – Putative pSTAT1::pSTAT2 binding elements (BE1 and BE2)

Yellow – pmESM1 QPCR probe binding sites for ChIP-QPCR

BE1 corresponds to pmESM1 probe-1 and BE2 corresponds to pmESM1 probe-2

Supplementary Figure S6.

The P3 fragment of the ESM1 promoter contains two putative BEs. The P3 fragment sequence is as shown. Two putative BEs are in orange and green. Yellow highlights the sequences recognized by the pmESM1 QPCR probe used in the ChIP experiments.

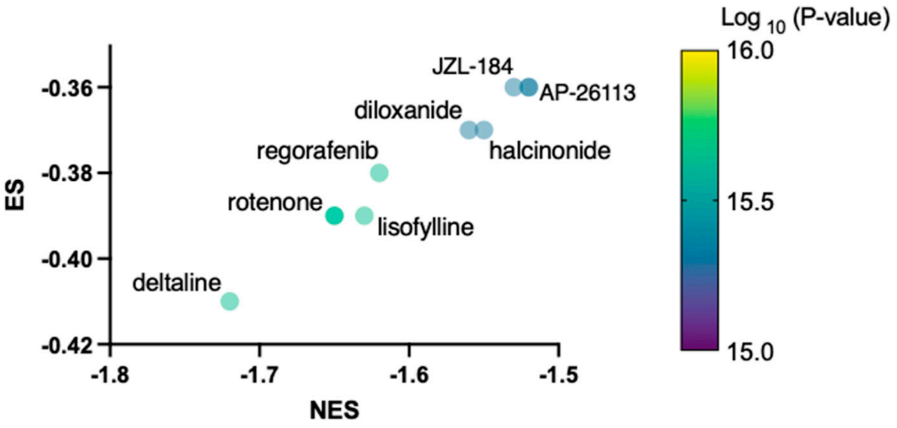

Supplementary Figure S7.

Identification of potential IRF5 inhibitors using Connectivity Map (CMap) analysis: The scatter plot shows the Enrichment Score (ES) and Normalized Enrichment Score (NES) for various compounds, indicating their potential inhibitory effects on IRF5-related gene expression. Each point represents a compound, with its position determined by ES (y-axis) and NES (x-axis) values. The color gradient corresponds to the –log10(p-value), indicating the statistical significance of the association between the compound and the gene expression signature, with darker colors representing higher significance. Notable compounds with potential inhibitory effects include JZL-184, AP-26113, and halcinonide.

Supplementary Figure S8. ESM1 Full-Length Promoter Sequence

>NC_000005.10:c54987593-54985494 Homo sapiens chromosome 5, GRCh38.p14 Primary Assembly

P3

TGGTAATCTACATAAACTGCTTAGCATGGCATCTGGCACACAGTCAGATCTCTGTAAAGGTTAGATATTATTATTAA
CCACTATTCCAGTTAACTACTCCTCAGAAAGTGGTGTTCAGTAACAAGGGACATTTTCTAGGGTCAGCCTAAATTG
CGACCTAGGCCATTGGAACTATTGGCCATGAAAGCACAATTATGTATTGTAATAGGCAATAGAATATGTGGCTCT
GACATTAGACTAACATCCATCCCTTCAATATAGGGACCTTAGGCAAATTAACCTCTTTCTTACTCAGTTTCTTCTTTT
AAAAAATAAGCATGATAATAGCAGCCACTTCATAATATTGTGATGAGGTGCAAATGAGTTAATGCATGTAAAGTGTT
TAAGCTATTAACTCAACAAATATTACCCATTACTGAAAACATCAGAGTGTGCCACCTGCTAAGTAAGTGCTTCTGG
GGAGATAGAGTATTTCAAATTGGATTGTCATGAAAAATTTGGAAAATTTGAAAGCTAAAACTCTTGCTTTCATTTCCT
ACTGCCTCAATTCTCTGTATTCTGAATTTACTCCCTGTGTCCCCCTAAAATTGCATTTGCAAAAGCCGATCTTCAA
GTTACATCCAATGCCCGCTCTGCCTCATCTTCTATGGGAAACAAGAATTTTAGAGGTCAGGTAGCCTAACACCAT
CAATTCTCAAAAGAGGAAGCTGAGGCCAAGAGAAGTCCTGTGAATT

P2

CAGGTAGCCTAACACCATCAATTCTCAAAAGAGGAAGCTGAGGCCAAGAGAAGTCCTGTGAATTTCTTACAGCT
CATTTGTGACAGACCAAGAATTACCCACTTTACTGGGTTGTTATTTACTAAGTGACAGTGAGTCTATATCTCTTTTGA
CAAGTGAGGTGGGGGCATGGAATTCGGCATGTGGTTGGTGTAAGAACTCCCCTCTCTCCTCTTTAACCTTACTTA
ATAAGACCCTGGCACAGTTGATATTTTAAGAGGGCTACTCTGTTTTCCCAGAGGGACCTAGGCACGGTAACCCT
CTTAGCATGCAGACCTTGTTTCCTGAGGGGTAATTGTTTCCCTTCCCTGTGACTTGTTTCTTGGGGGCTGTGTTCTG
ATTTTCCTGCTGAGCCACTTGTTGCCTTGGGCTGGCTGCCGCGCTTGGCAGTTTTTAGTGAGGGCTCTGATAGAT
GCCAGGAGGTGAGGGGAAGGGCTCTGGGTGGACTCCGTCATTGGACAAGCAGACTTAGTGATGGATGAGCCT
TCCCCTGAGGAAGTTTTGGATCAGAAGTCCAACTGATAAGTTTTTCCAGAATTGAGTAACCCAGAAGCAGTGCCG
AAAGGATCTTACCTCTCTTGTGGCTTTTTGTATTGATTTTAAAAGAAATTCTCAGAGGCAGTTCCACATTGTACTGGA
AGCACAGCTATATCCACAATAGGCTTAGATATATGTAACATGA

P1

AGGCAGTTCCACATTGTACTGGAAGCACAGCTATATCCACAATAGGCTTAGATATATGTAACATGAATTGCTTTAG
AAATAACATTTGAGGAGAGGGGTGAGAGGAAGGAAGAGAGGGTCTTAAAAAATAGCCCTATCAAAATATTTTCTTT
CTTCTAAGTATTGAAAAGACACAATATAACCCTTTCTTCTTTCAAATGATCTCATAGCTATTTGTTGAGGGGAAATAC
CAAATGTTTATTATTTTTTTTGAAGAAGCTTCTTCGGTCCTGATGATTCATGTTGATATCATTTTCCTCCTGACTACAG
AGGCTCTGAGACAAAGCTACACCTCAAGTGATATGCCAGGGTCAGAACAATTCCCGTCCTGAAGGAGGGTGTG
CAACCTTCTTTATCCCTCCTTCACAGACGTCCTTGAGCCCTTGAGACGGATGTGAGTGAGTTTTTCAGTCCTCAT
GCAAAACAACCATCTAAACATAACAGATGACATCAGCTTGGGCTTTTCAATTCCTGGATGGCAGCAGCGTGTTAA
TCCAGCCTTCATCCTGGATTTCATAAACCAAAACAAGAGAGCCTGGCAGGAGGACAGCGCTGCTGCTGGGTTG
AGGAAATTGATGACGGGAAAGCATGCGGGCAACCCAGTGTATAAAACTCATAAACGTGTAGGCAGAGGCTCAG
CTACCAGTTTGGACGGCTGCTTCCCACCAGCAAAGACCACGACTGGAGAGCCGAGCCGGAGGCAGCTGGGA
AACATGAAGAGCGTCTTGCTGCTGACC
